# Towards the new normal: Transcriptomic convergence and genomic legacy of the two subgenomes of an allopolyploid weed (*Capsella bursa-pastoris*)

**DOI:** 10.1371/journal.pgen.1008131

**Published:** 2019-05-13

**Authors:** Dmytro Kryvokhyzha, Pascal Milesi, Tianlin Duan, Marion Orsucci, Stephen I. Wright, Sylvain Glémin, Martin Lascoux

**Affiliations:** 1 Plant Ecology and Evolution, Department of Ecology and Genetics, Evolutionary Biology Centre and Science for Life Laboratory, Uppsala University, Uppsala, Sweden; 2 Department of Ecology and Evolutionary Biology, University of Toronto, Toronto, Canada; 3 CNRS, Univ. Rennes, ECOBIO [(Ecosystèmes, biodiversité, évolution)] - UMR 6553, Rennes, France; University of California Davis, UNITED STATES

## Abstract

Allopolyploidy has played a major role in plant evolution but its impact on genome diversity and expression patterns remains to be understood. Some studies found important genomic and transcriptomic changes in allopolyploids, whereas others detected a strong parental legacy and more subtle changes. The allotetraploid *C. bursa-pastoris* originated around 100,000 years ago and one could expect the genetic polymorphism of the two subgenomes to follow similar trajectories and their transcriptomes to start functioning together. To test this hypothesis, we sequenced the genomes and the transcriptomes (three tissues) of allotetraploid *C. bursa-pastoris* and its parental species, the outcrossing *C. grandiflora* and the self-fertilizing *C. orientalis*. Comparison of the divergence in expression between subgenomes, on the one hand, and divergence in expression between the parental species, on the other hand, indicated a strong parental legacy with a majority of genes exhibiting a conserved pattern and *cis*-regulation. However, a large proportion of the genes that were differentially expressed between the two subgenomes, were also under *trans*-regulation reflecting the establishment of a new regulatory pattern. Parental dominance varied among tissues: expression in flowers was closer to that of *C. orientalis* and expression in root and leaf to that of *C. grandiflora*. Since deleterious mutations accumulated preferentially on the *C. orientalis* subgenome, the bias in expression towards *C. orientalis* observed in flowers indicates that expression changes could be adaptive and related to the selfing syndrome, while biases in the roots and leaves towards the *C. grandiflora* subgenome may be reflective of the differential genetic load.

## Author summary

Most plant species have a polyploid at some stage of their ancestry. Polyploidy, genome doubling through either multiple copies of a single species or through genomes of different species coming into the same nucleus, is therefore a crucial step in plant evolution. Understanding its impact on basic biological functions is thus a matter of interest. Shepherd’s purse (*Capsella bursa-pastoris*) is a major weed that appeared about 100,000 years ago through hybridization of two diploid species of the same genus. In the present project, we measured genetic diversity and analyzed gene expression patterns in flowers, roots, and leaves of *C. bursa-pastoris* individuals as well as in its two parental species, the outcrossing *C. grandiflora* and the self-fertilizing *C. orientalis*. Our data shows that, after 100,000 generations of evolution, the origin of the two subgenomes can still be seen: the genome inherited from *C. grandiflora* still differs from the one inherited from self-fertilizing *C. orientalis*. However, there are also signs that the two genomes have started to work together and are jointly regulated, and the way expression pattern varied across the three tissues indicates that the evolution of gene expression was adaptive.

## Introduction

Polyploidy, and in particular allopolyploidy, whereby a novel species is created by the merger of the genomes of two species, is considered to be a common mode of speciation in plants [1] as it induces an instant reproductive isolation, the difference in chromosome number impeding reproduction with the parental species. In the case of allopolyploidy, the daughter species thus has two divergent subgenomes at inception, one inherited from each parental species. Such an increase in genome copy number can be advantageous and could partly explain the apparent evolutionary success of allopolyploid species ([2, 3] but see [4]). For instance, genome doubling creates genetic redundancy, thereby increasing genetic diversity and allowing the masking of deleterious mutations through compensation. Genome doubling and initial redundancy also offer new possibilities for the evolution of genes over time: one copy can degenerate, both can be conserved by dosage compensation [5] or their pattern of expression can diverge and even lead to the evolution of new functions (see [6] and references therein). Gene redundancy also potentially allows tissue-specific expression of different gene copies [7, 8]. On the other hand, the evolutionary success of allopolyploids can also appear paradoxical since the birth of a new allopolyploid species will also be accompanied by numerous challenges [9–12]. These challenges are first associated with the initial hybridization between two divergent genomes, implying, among other things, potential changes of gene expression patterns [13].

The magnitude of gene expression changes has been reported to vary substantially across polyploid species, from minor modifications [14, 15] to so-called “transcriptomic shock” [8]. The balance in expression pattern between the two subgenomes also seems to be highly variable and ranges from the additivity of parental expression to extreme non-additivity. Several forms of non-additivity have been widely observed, such as homeologue expression bias, when the relative expression contributions from the two homeologues are altered, and expression level dominance, when the total expression level of both homeologues is similar to only one of the parental species [16, 17] (see [18] for definitions). These patterns also evolve through time. For example, in *Mimulus peregrinus* the genome-wide homeologue expression bias was established early on but also increased over successive generations [19]. However, the generality, timing, and causes of changes in expression pattern of the two parental genomes remain poorly known beyond a few case studies [17, 20] and may, to a large extent, depend on parental legacy because a part of the observed differences between the two subgenomes of the allopolyploid species may have already been present between the parental species [3].

Ultimately, changes in patterns of gene expression will follow from modifications in gene expression regulation. Differences in gene expression can be due to changes in *cis*- and *trans*-regulatory elements. *Cis*-regulatory elements alter allele-specific expression and are generally located close to the gene they regulate (e.g., promoters), whereas *trans*-regulatory elements can affect both alleles and can be located anywhere in the genome [21–24]. In the case of a newly formed allopolyploid species, one would expect the two copies of a gene to be under the influence of *trans*-regulatory elements inherited from both parents and its expression level to first move towards the mean expression of the two parental species. Retaining the parental pattern of expression in each subgenome would imply that only *cis*-regulation takes place, or there are forces opposing the establishment of cross *trans*-regulation. For instance, one could expect purifying selection to have a larger impact on *trans*-acting mutations than on *cis*-acting ones because the former have more pleiotropic expression than the latter. If so, the residual variants will mostly be *cis*-acting ([25] but see [26]). It was also shown that a gene is often under the influence of both *trans*- and *cis*-regulatory elements that act in opposite directions [24], leading to a *cis*-*trans* compensation that prevents overshooting optimal overall expression level. Such compensation between *cis*- and *trans*-regulatory elements is one of the predictions of the enhancer runaway (ER) model proposed by Fyon et al. [27]. Under the ER model, and especially in outcrossing species where heterozygotes are frequent, *cis*-regulatory variants facilitate the exposure of alleles to purifying selection. If the enhancer and the gene they regulate are linked then the up-regulating variants will hitch-hike with the allele carrying the lowest number of deleterious mutations, leading to an open-ended escalation in enhancer strength [27]. As selection on expression appears to be primarily stabilizing [24, 28, 29], at least at intermediate evolutionary timescales [30], a compensatory effect of expression in *trans* is predicted [27, 31]. The relative importance of *cis*- and *trans*-regulation can be examined by comparing the relative expression in the parental species with the relative expression of homeologous genes in the newly formed tetraploid [21, 32, 33].

Differential expression between the two genomes could result from a differential accumulation of deleterious or slightly deleterious mutations between the two subgenomes or, alternatively, be also related to phenotypic or adaptive changes associated to the differences between the two parental species. If the differential expression is *only* due to differential accumulations of deleterious mutations, we would expect to see the same differential expression pattern across different tissues, whereas if differential expression is related to phenotypic or adaptive changes then we may expect to see differences depending on the tissue considered.

**Fig 1:**
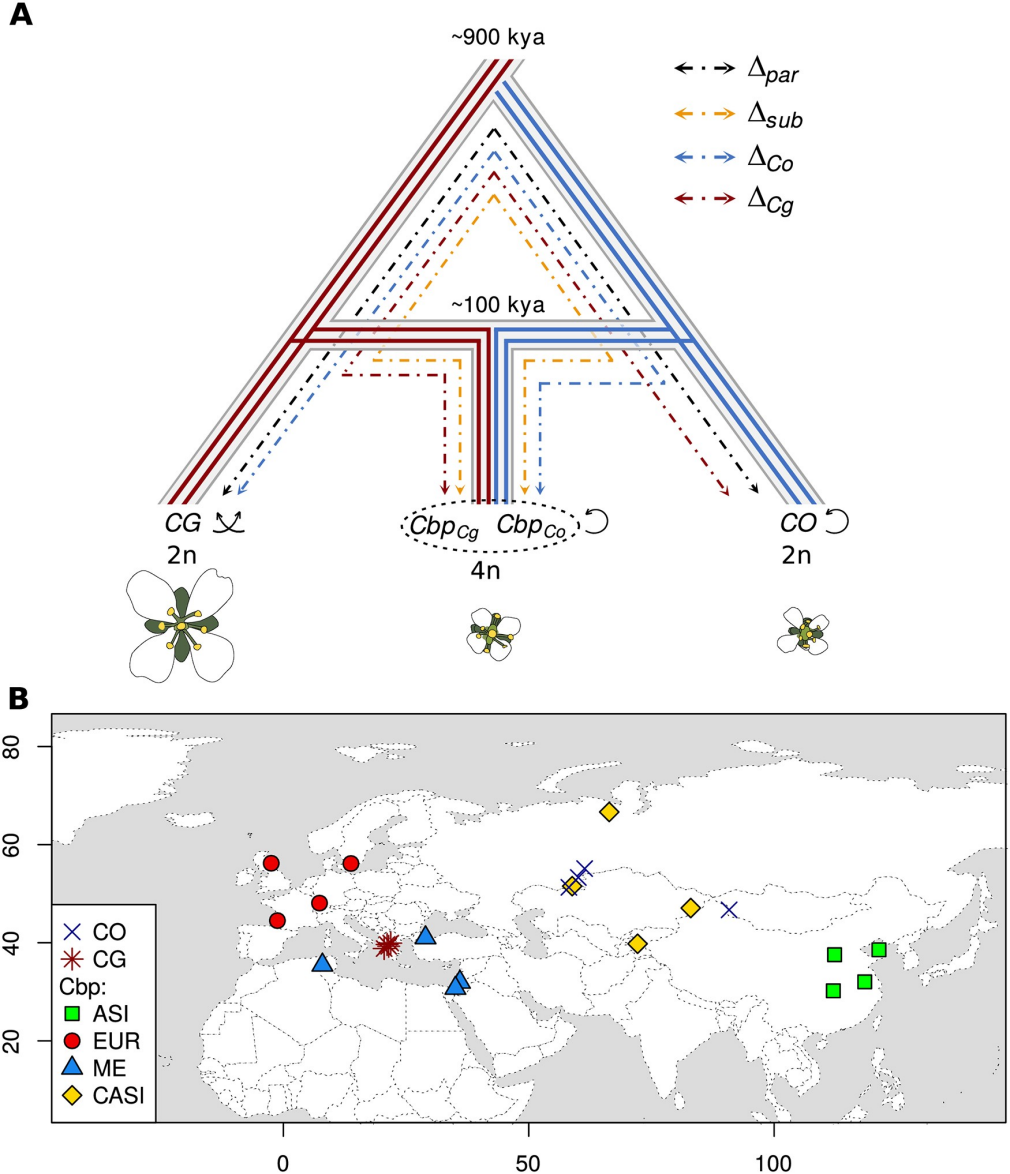
Evolutionary history and sampling locations of the three *Capsella* species used in this study. **A** Solid lines represent subgenomes segregation after the hybridization between *C. grandiflora* (*CG*) and *C. orientalis* (*CO*) ancestors. *C. grandiflora* and *C. orientalis* genetic backgrounds are marked with red and blue respectively. The ploidy levels (n) and the reproductive system are also indicated. Dashed and dotted lines represent the comparisons used to compute the gene expression convergence index (see Material and methods). **B**. CO, CG, ASI, EUR, ME, CASI correspond to *C. orientalis*, *C. grandiflora*, and four populations of *C. bursa-pastoris*, *Cbp*, (Asia, Europe, Middle East, and Central Asia) respectively. We shifted slightly population geographical coordinates when those overlapped to make all of them visible on the map.

Shepherd’s purse, *C. bursa-pastoris*, is an allotetraploid selfing species that originated some 100-300 kya from the hybridization of the ancestors of *C. orientalis* and *C. grandiflora* [15] (Fig 1A). The two parental species are strikingly different: *C. orientalis*, a genetically depauperate selfer, occurs across the steppes of Central Asia and Eastern Europe [34], whereas *C. grandiflora*, an obligate outcrosser with a particularly high genetic diversity, is primarily confined to a tiny distribution range in the mountains of Northwest Greece and Albania [34] (Fig 1). Among *Capsella* species, only *C. bursa-pastoris* has a worldwide distribution [34], some of which might be due to extremely recent colonization events associated with human population movements [34, 35]. In Eurasia, the native range of *C. bursa-pastoris* is divided into three genetic clusters—Asia, Europe, and the Middle East (hereafter ASI, EUR and ME, respectively)—with low gene flow among them and strong differentiation both at the nucleotide and gene expression levels [35, 36]. Reconstruction of the colonization history suggested that *C. bursa-pastoris* spread from the Middle East towards Europe and then expanded into Eastern Asia. This colonization history resulted in a typical reduction of nucleotide diversity with the lowest diversity being found in the most recent Asian population [35]. It has been possible to phase the subgenomes by assigning each genome sequence (or transcript) to a parental species sequence [37]. In stark contrast to many other studies of allopolyploids, such as maize, cotton, *Brassica*, *Xenopus laevis*, [38–44], the phased data suggested that the differences in deleterious variants between the two subgenomes of *C. bursa-pastoris* are largely a legacy of the differences between the two parental species and that biased fractionation, the biased loss of ancestral genomes in an allopolyploid, is limited [15, 45].

The aim of the present study was to address questions on the evolution of gene expression patterns of the two subgenomes of the allotetraploid shepherd’s purse *C. bursa-pastoris* since they derived from the two parental species. We focused on two main questions. First, has the relative contribution of *cis*- and *trans*-regulation been altered by polyploidization? Second, could differential expression between the two subgenomes only results from a differential accumulation of deleterious/slightly deleterious mutations (nearly neutral hypothesis) or is it *also* related to phenotypic differences between the two parents (adaptive hypothesis)? One parent is outcrossing (*C. grandiflora*) and has large flowers as it needs to attract pollinators while the other parent is self-fertilizing (*C. orientalis*) and has tiny flowers. Hence one may expect differential expression in flower tissues of selfing *C. bursa-pastoris* to be biased towards the *C. orientalis* expression levels under the adaptive hypothesis whereas tissues that have not experienced adaptive specialization might show an expression bias towards *C. grandiflora*.

To address these questions and, more generally, to characterize the expression pattern of *C. bursa-pastoris*, we analyzed the genomes and the transcriptomes of three tissues (flowers, leaves, and roots) of 16 accessions coming from different populations of the *C. bursa-pastoris* natural range and compared them with those of the parental lineages *C. grandiflora* and *C. orientalis* (four accessions each) (Fig 1). In total, 24 transcriptomes in three tissues and 24 genomes were analyzed.

One hundred thousand generations after its inception, *C. bursa-pastoris* does not show any sign of a transcriptomic shock. Instead, our data revealed highly concerted changes with the expression levels of the two subgenomes converging towards an intermediate value. This was achieved by a balance between *cis*-and *trans*-regulation and a strong parental legacy that was also observed for the accumulation of deleterious mutations over the two subgenomes. While the differential accumulation of deleterious mutations between subgenomes could explain part of the differential expression between them, there were also significant tissue-specific differences in subgenome dominance and convergence indicating that adaptive changes may also have contributed to the evolution of the expression patterns of the two subgenomes.

## Results

### Population genetic structure

**Fig 2:**
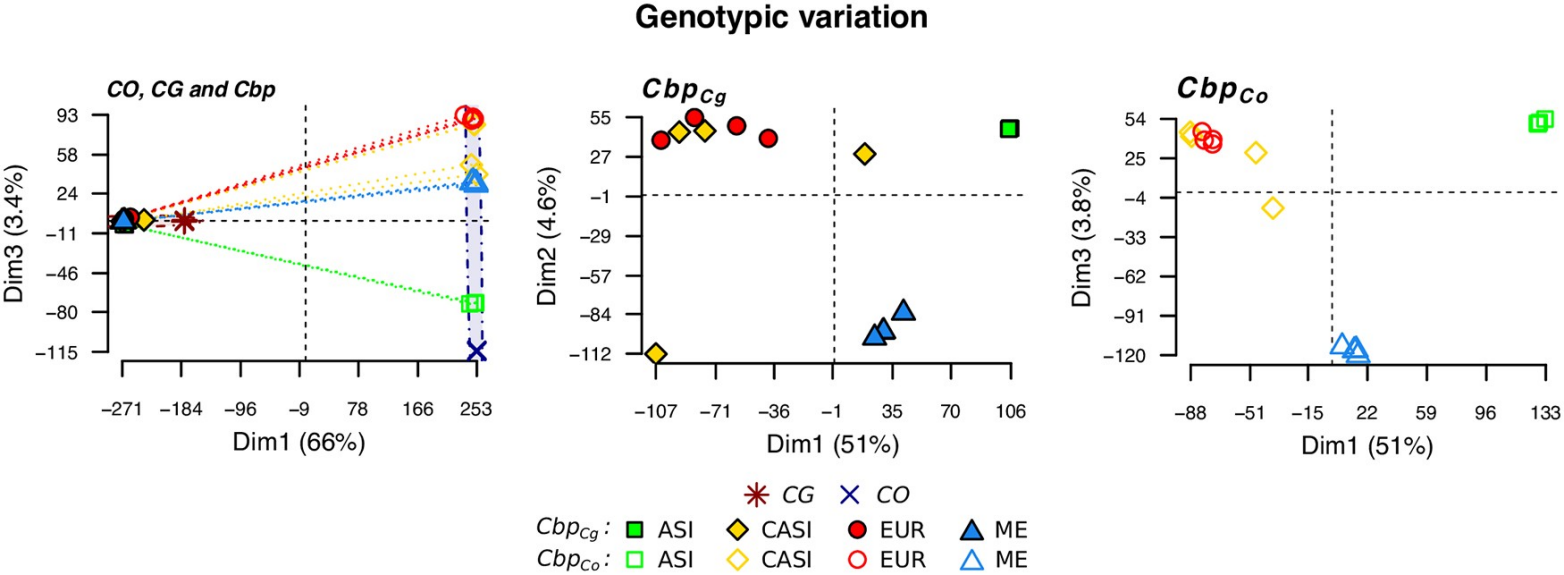
Genomic variation patterns in three *Capsella* species. Variation was visualized with principal component analyses based on the SNPs of *C. grandiflora* (*CG*), *C. orientalis* (*CO*), and four populations of *C. bursa-pastoris* (*Cbp*) (Asia (ASI), Central Asia (CASI), Europ (EUR), and Middle East (ME)). The left plot shows variation in the three species with lines connecting subgenomes of corresponding *Cbp* accessions and the dash-dotted circles highlighting two subgenomes of *Cbp*. The middle and right plots show only the variation within the subgenomes of *C. bursa-pastoris* (*Cbp*_*Cg*_ and *Cbp*_*Co*_).

In order to assess the relationship of the newly obtained Central Asian samples with other populations, we analyzed the population structure of our samples. A SNP-based PCA (670K genomic SNPs without any missing data) confirmed the phylogenetic relationships between *C. grandiflora* (*CG*), *C. orientalis* (*CO*), and *C. bursa-pastoris* (*Cbp*) described in [35–37]. The first principal component (Dim1) explained the majority of the variance (66%) and clearly discriminated *CG* and the *Cbp*_*Cg*_ subgenome from *CO* and the *Cbp*_*Co*_ subgenome (Fig 2, left panel). To investigate further population structure within *C. bursa-pastoris*, we then focused on genetic variation in each subgenome (Fig 2, middle and right, respectively for *Cbp*_*Cg*_ and *Cbp*_*Co*_). In both cases, there were three main clusters gathering accessions from Europe (EUR), Asia (ASI), and the Middle East (ME), respectively. Accessions from Central Asia (CASI) tended to cluster with European accessions for both subgenomes, even if they were more scattered. A phylogenetic analysis also confirmed that the new samples from Central Asia were most similar to the European genetic cluster and showed that they did not form a separate genetic cluster (S1 Fig).

### Global variation in gene expression reflects genetic relationships

Given that the gene expression patterns in homeologue-specific and total expression can produce different results [46], we performed a differential gene expression analyses on both the unphased and phased data. Pairwise comparisons of a number of differentially expressed (DE) genes between species in unphased data (16,039 genes) showed that patterns of expression varied across tissues. First, the number of differentially expressed genes between parental species was the highest in flower tissues, while leaf tissues were the least differentiated (S2 Table). Second, in flowers, overall gene expression of *C. bursa-pastoris* was the closest to *C. orientalis*, while in the two other tissues it was the closest to *C. grandiflora* (S2 Table). At the population level, no clear pattern appeared: for instance, ME accessions were the closest to *C. grandiflora* in roots, while ASI accessions were the closest to *C. grandiflora* in leaves and CASI accessions in flowers (S3 Table).

**Fig 3:**
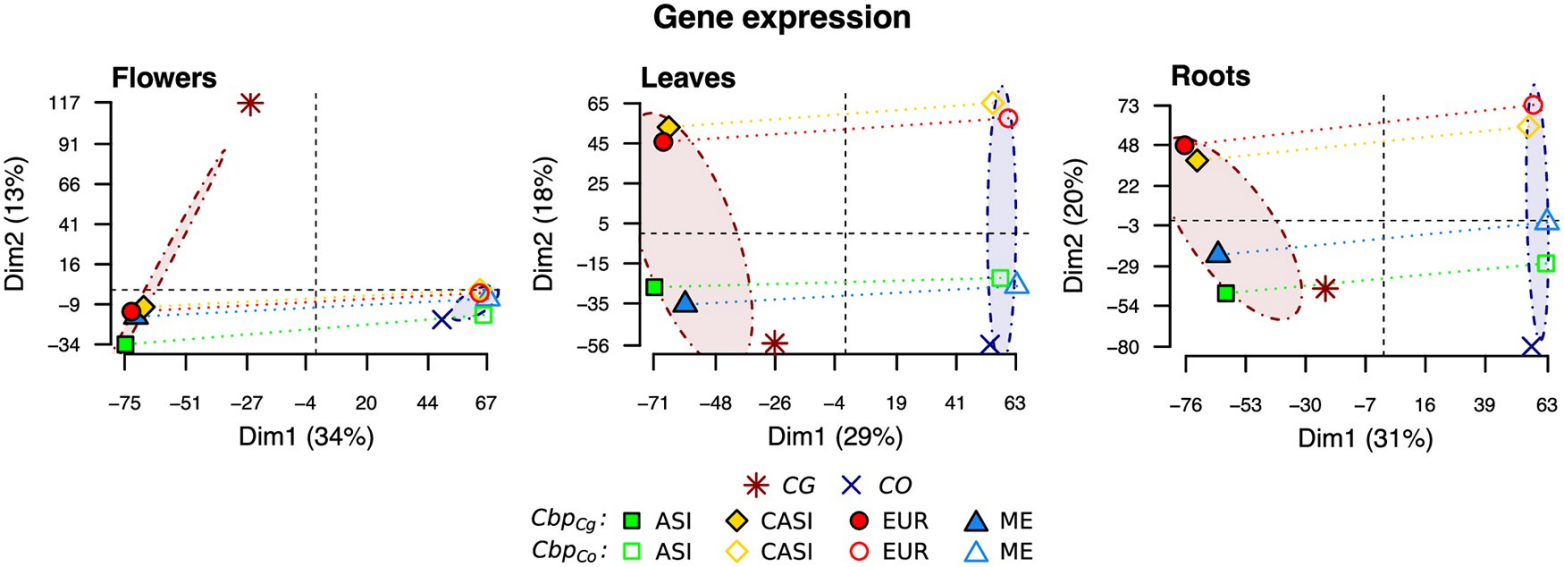
Transcriptomic variation patterns in three *Capsella* species. Variation was visualized with principal component analyses of phased gene expression data (11,931 genes) for the three different tissues. CO, CG, ASI, EUR, ME, and CASI correspond to *C. orientalis*, *C. grandiflora*, and four populations of *C. bursa-pastoris*, *Cbp*, (Asia, Europe, Middle East, and Central Asia), respectively. The dash-dotted circles highlight the two different subgenomes of *Cbp*.

Gene expression variation was then surveyed in 11,931 genes for which phased expression of the two subgenomes was available in all populations of *C. bursa-pastoris*. Clustering of population/species mean expression values confirmed that the main difference in overall expression variation was between tissues (S2 Fig). The principal component analyses of the three tissues separately (Fig 3) revealed that the global variation pattern in gene expression reflected phylogenetic relationships (Fig 3 and S3 Fig). The two subgenomes of *C. bursa-pastoris* were most similar to their corresponding parental genomes along the first principal component, Dim1, *i.e*. expression in the *Cbp*_*Cg*_ subgenome grouped with *C. grandiflora*, and the *Cbp*_*Co*_ subgenome grouped with *C. orientalis*. The second principal component, Dim2, reflected population structure; here again CASI accessions grouped with EUR accessions.

Testing for homeologue-specific expression (HSE) in *C. bursa-pastoris* showed that on average 4,096 genes (∼34%) per sample were significantly differentially expressed between the two subgenomes (*FDR* < 0.05). The expression ratio between subgenomes (defined as $\frac{Cbp_{Co}}{Cbp_{Co}+Cbp_{Cg}}$) was on average 0.496 across all genes and 0.493 across genes with significant HSE indicating no strong bias towards one of the subgenomes (S4 Table). The ratio in DNA reads was 0.497 and thus there was no strong mapping bias towards either subgenome. Analyses of differential expression revealed no bias in the number of differentially expressed genes toward one subgenome either when comparing tissues (S5A Table, flowers and leaves being the most differentiated tissues and leaves and roots the least) or *Cbp* populations (S5B Table, Middle East and Asia being the most distant, except for *Cbp*_*Co*_ in flowers, while Europe and Central Asia are the closest).

### Strong parental legacy and both *cis*- and *trans*-regulatory changes

**Fig 4:**
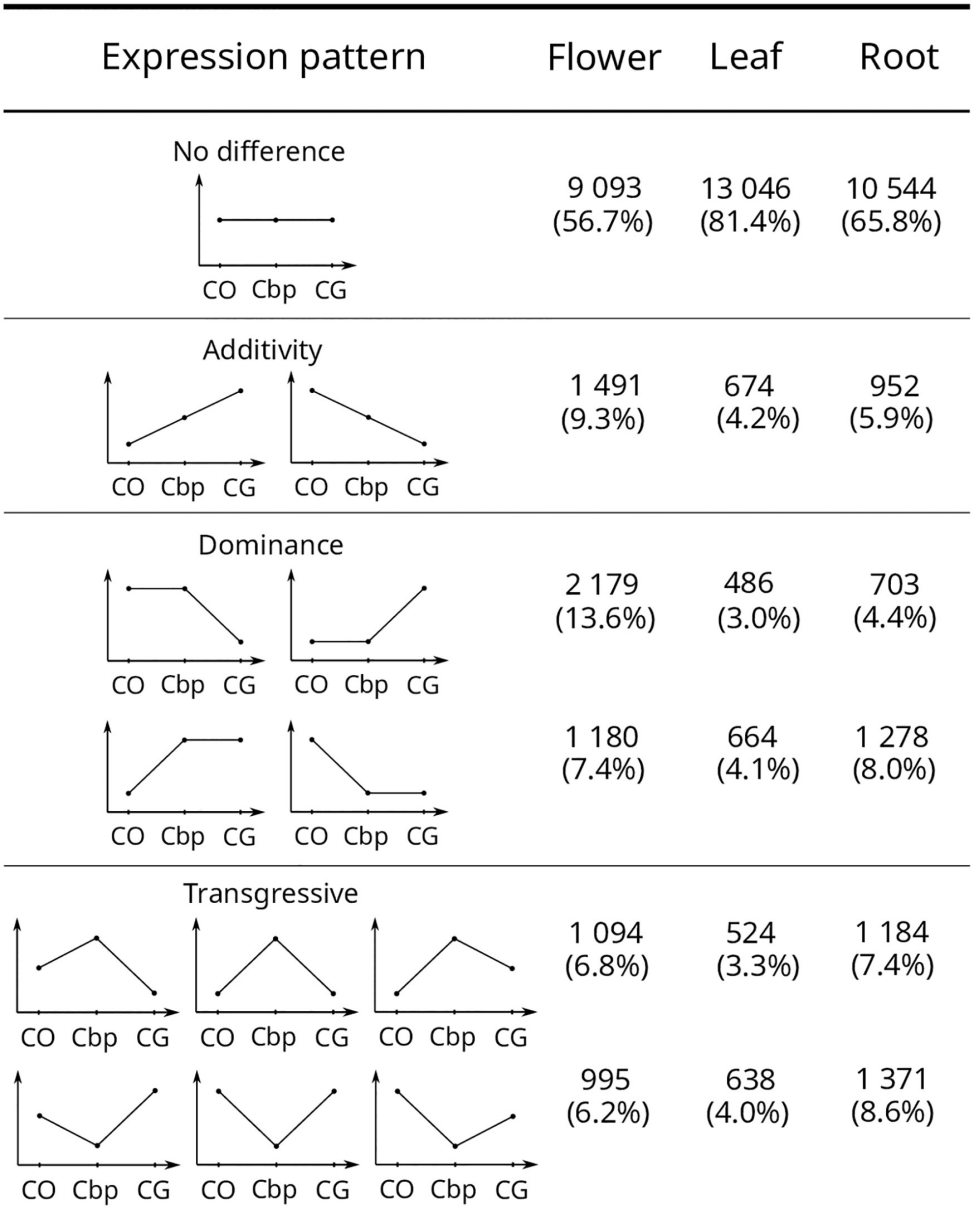
Levels of gene expression in *C. bursa-pastoris* relative to its parental species. CO, CG, and Cbp correspond to *C. orientalis*, *C. grandiflora*, and *C. bursa-pastoris*, respectively. The y-axis indicates the level of expression. Expression levels were considered significantly different for the *FDR* < 0.05. In total, 16,032 genes were analyzed.

In order to investigate the total expression level changes in *C. bursa-pastoris* after *C. grandiflora* and *C. orientalis* hybridization, expression patterns of unphased data across the three species were classified into four categories: *No difference*, *Intermediate/Additivity*, *Dominance* and *Transgressive* (Fig 4). Up to 55-80% of the genes in *C. bursa-pastoris* were expressed at the same total level as in the parental species and 5 to 10% showed levels of expression intermediate to that of parental species. The dominance of one parental species over the other was most evident in flowers and roots. In flowers, ∼14% of *C. bursa-pastoris* genes were expressed at the same level as in *C. orientalis* but differed significantly from *C. grandiflora*, and ∼8% were expressed at the same level as in *C. grandiflora* but at a different level than in *C. orientalis*. The opposite dominance pattern was detected in the root tissue. Finally, a transgressive expression pattern, when expression levels in *C. bursa-pastoris* exceeded or were lower than the expression level of both parents, was detected in 8-16% of genes.

**Fig 5:**
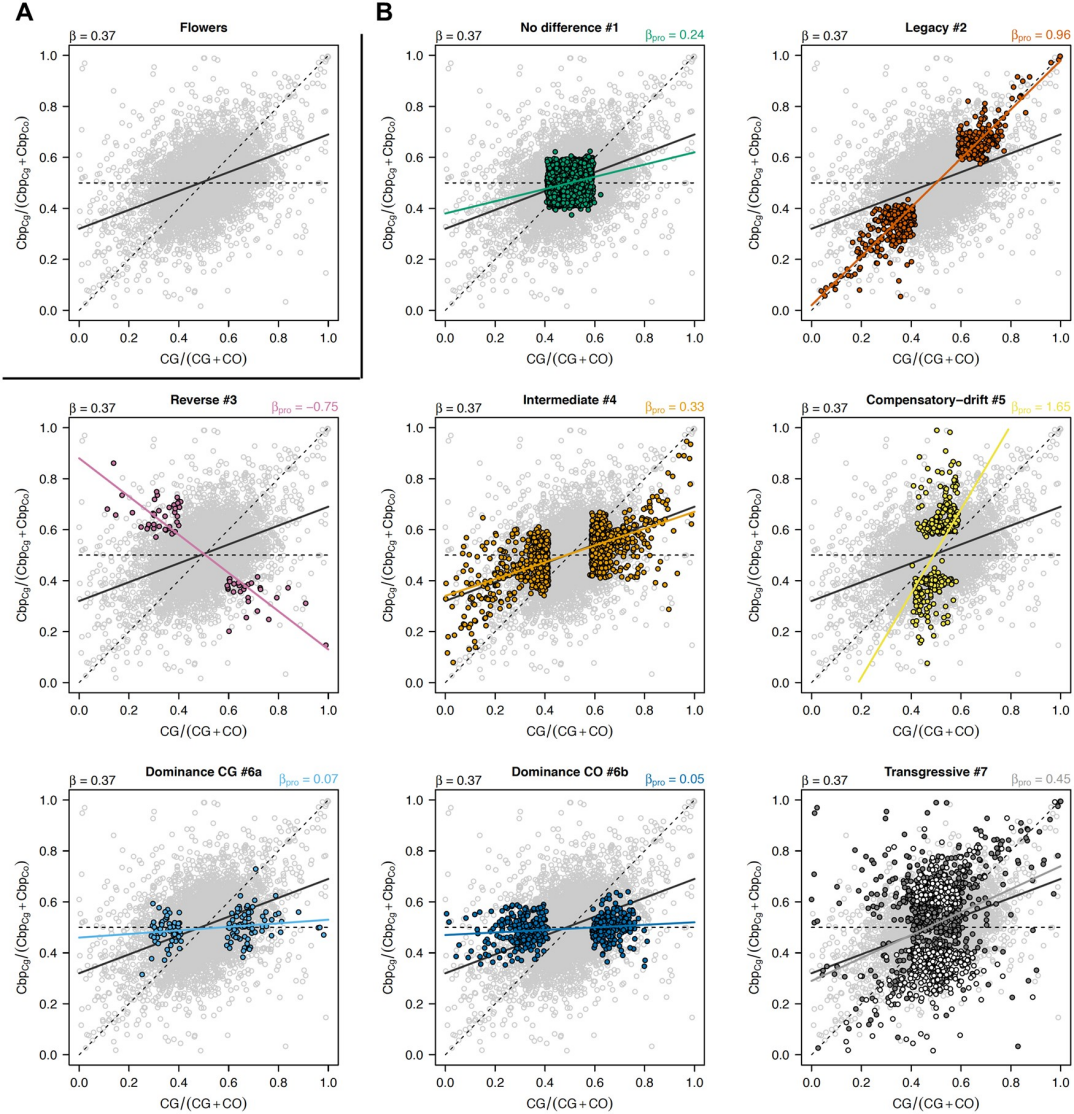
Relationships between the relative expression of the *C. bursa-pastoris* subgenomes and the relative expression of parental species. The figure shows expression in flower as an example. **A**. Top-left panel is for all transcripts (11,931). **B**. Transcripts belonging to a specific category. The diagonal dashed lines indicate 100% *cis*-regulation divergence while the horizontal dashed lines indicate 100% *trans*-regulation. The solid lines give the slopes of the linear regressions between both ratios either for all transcript (black) or for transcript belonging to a specific category. *β* is the slope of the corresponding regression. For *Transgressive* category (bottom right panel), dark gray corresponds to categories #7a and b, light grey is for category #7c (see Fig 6).

Gene expression in *C. bursa-pastoris* was further investigated by assessing the relative importance of *cis-* and *trans*-regulatory elements. The expression ratio of the two subgenomes was compared to the expression ratio between the two parental species (Fig 5A). For a given gene, if its expression in the homeologous genes of *C. bursa-pastoris* is only regulated by *cis*-regulatory changes, it should be completely explained by the divergence between the parental species (the diagonal line in Fig 5A). On the other hand, if homeologous genes are equally expressed in *C. bursa-pastoris* but not in the parental species, this means that *Cbp* expression is mainly controlled by *trans*-regulatory elements (the horizontal line in Fig 5A) [21]. First, the relationship between expression ratios in *C. bursa-pastoris* and parental species was positive and highly significant for all three tissues (*p* < 0.001), and the slope was intermediate between what would be expected if there were either only *cis*-(*β* = 1) or only *trans*-regulatory (*β* = 0) changes (*β* = 0.37, 0.42 and 0.46, respectively for flowers, leaves and roots). This indicates a strong parental legacy effect in expression of the two subgenomes of *C. bursa-pastoris* and suggests a joint effect of *cis*- and *trans*-regulation. Second, the variance of the expression ratio between subgenomes was significantly smaller than the variance of the expression ratio between parental genomes (Fisher’s variance test, all *p* < 0.001), indicating that the two subgenomes are closer to each other than the parental genomes are, therefore supporting a co-regulation of the two subgenomes through a mixture of *trans*- and *cis*-regulation [21, 32]. Finally, the slope of the regression between the two expression ratios was the weakest in flowers, suggesting a slightly stronger *trans*-regulation and a higher level of constraints in this tissue than in roots and leaves [32].

### Classification of expression patterns

**Fig 6:**
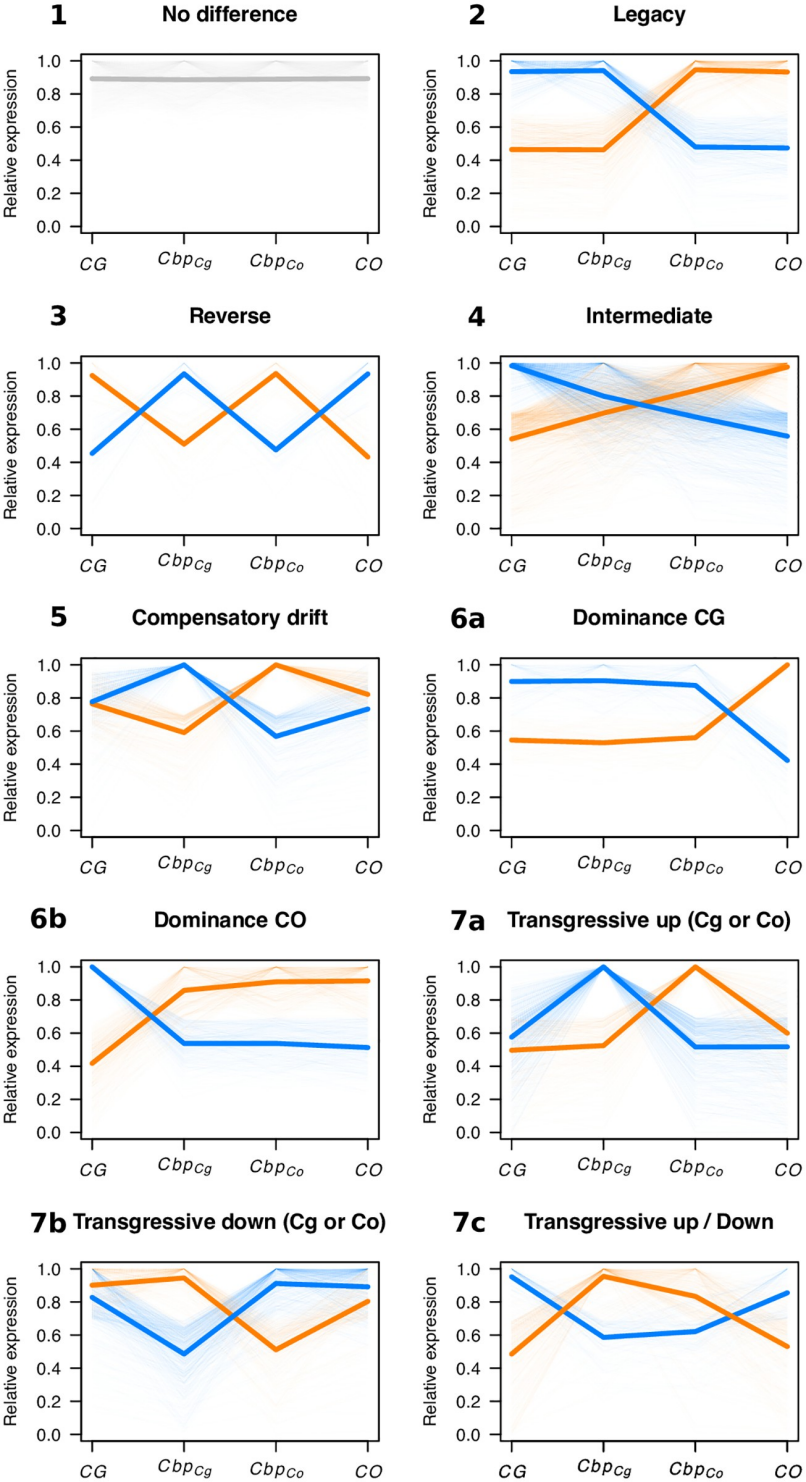
Main categories of expression variation of *C. bursa-pastoris* subgenomes relative to expression in parental species. The figure shows expression in flower as an example. Each transcript was assigned to one of seven main categories defined from the relative expression pattern of *Cbp* subgenomes (*Cbp*_*Cg*_ and *Cbp*_*Co*_) and parental species (*CG* and *CO*). For each category, dashed lines correspond to single transcript relative expression to the maximal expression of this transcript in parental genomes or subgenomes. Solid lines indicate the average expression for each genome or subgenome. Colors discriminate alternative patterns in the same category.

As mentioned above, subgenome expression level relative to parental species expression can help to disentangle the role of *cis*- and *trans*- components on overall gene regulation. However, the comparison between the ratios of expression in the tetraploid and in the parents is not sufficient to distinguish all possible patterns. We thus classified the expression patterns at the equivalent developmental stage in genes from the two subgenomes and the parental species in seven main categories by comparing the four expression levels (see Fig 6 for an example with flower tissues). The majority of the transcripts was not differentially expressed between parental genomes and subgenomes (*No difference* category), ranging from ∼60% in flowers to ∼78% in leaves (Table 1). However, the slope of the regression between relative expression of subgenomes and parental species clearly indicated that, even if the expression levels were not significantly different between parental species and *C. bursa-pastoris* subgenomes, crossed *trans*-regulation tended to make the two subgenomes expression closer to each other than to either parental species (Fig 5B “*No difference*” and Table 1). About 9% of genes had an *Intermediate/Additive* expression, *i.e*., the expression of both subgenomes being in between the expression of the two parental species. As expected this pattern was due to a combination of both *cis-* and *trans*-regulation (*β* ≃ 0.3 − 0.4). Only 3% showed a strict *legacy* of parental species expression which is primarily due to *cis*-regulation (*β* ≃ 1). About 4% of the genes showed a *Dominance* pattern of either *CG* or *CO* parental genetic background (i.e., both subgenome expression are similar to that of one parental species, categories 6a and 6b, Fig 6). However, within transcripts showing a *Dominance* pattern, 76% of the transcripts showed a dominance of *CO* in flowers, while there were only 45% and 34% in leaf and root tissues (Table 1). The *Dominance* pattern seems to be due to a dominance of transcription factors from one subgenome over the other (*β* ≃ 0.05 − 0.2); in favour of *CO* parental genetic background in flowers and of *CG* parental genetic background in leaves and roots (Fig 5B and Table 1). Finally, 3% of the genes had a *Compensatory-drift* profile (parental species expressions are similar but subgenome expressions diverge), a mere 0.4% showed a *Reverse* profile (each subgenome expression is similar to the opposite parental species) and about 10% of the transcripts showed a *Transgressive* pattern, either because of one (categories 7a and 7b) or of both subgenomes expression (category 7c) (Fig 5B and Table 1). These last profiles are less straightforward to interpret in terms of *cis*- and *trans*-regulation pattern as they involve more complex post-hybridization regulation processes.

**Table 1:** Expression variation of *C. bursa-pastoris* subgenomes relative to expression in parental species across different tissues. The percentage of transcripts within each category is given for all genes or only differentially expressed genes (*i.e*, without *No difference* category). The slope of the regression of relative expression between subgenomes and relative expression between parental species for all genes per category is also provided (*β*, see Fig 5). The percentages of transcripts showing a dominance of either *Cbp*_*Cg*_ or *Cbp*_*Co*_ are given in parenthesis.

Categories		Flowers			Leaves			Roots		
		Transcripts (%)			Transcripts (%)			Transcripts (%)		
		All	DE only	*β*	All	DE only	*β*	All	DE only	*β*
No diff.	1	60.4	-	0.24	78.4	-	0.32	67.6	-	0.32
Legacy	2	4.5	11.4	0.96	2.3	10.6	0.94	3.2	9.9	0.96
Reverse	3	0.5	1.3	-0.75	0.2	0.9	-0.78	0.4	1.2	-0.78
Intermediate	4	12.6	31.9	0.33	6.8	31.5	0.44	8.8	27.3	0.41
Comp. drift	5	3.8	9.7	1.65	1.5	6.9	1.52	2.8	8.7	1.81
Dominance	6a	1.2	3.0 (24)	0.07	1.7	7.9 (54)	0.18	2.7	8.4 (66)	0.14
	6b	3.8	9.6 (76)	0.05	1.4	6.5 (45)	0.11	1.4	4.3 (34)	0.1
Transgressive	7a	5.1	12.9	-	2.1	9.7	-	4.2	13	-
	7b	5.2	13.2	-	2.6	12	-	4.4	13.7	-
	7c	2.8	7.1	0.45	3	13.9	0.55	4.3	13.4	0.61
	Total	100	100	0.37	100	100	0.42		100	0.46

Finally, although the relative proportions of the different categories were globally conserved across tissues (Table 1), expression patterns of individual genes were strongly tissue-specific. In our data, only half of the genes showed the same expression pattern in all three tissues. The most conserved category was *No difference*, 77%, and the least conserved one was *Compensatory-drift*, 3%. Pairwise comparisons between tissues revealed that the number of genes for which the expression pattern changed from one tissue to another was the largest between flowers and roots tissues (42%) and the smallest between leaf and root tissues (33%).

To conclude, only about 10% of the 11,931 transcripts had a transgressive or a reverse expression pattern. Expression patterns were poorly conserved between tissues except for the *No difference* category, indicating that the evolution of expression regulation is highly tissue-specific. Flower tissue differed the most from the two other tissues. In addition to a lower proportion of differentially expressed genes, flower tissues also had the lowest proportion of *Transgressive* category in the differentially expressed genes, indicating that when expression changes occurred, they either took place within the expression range of the parental species or they were compensated by the other subgenome (*Compensatory-drift*). This suggests a higher level of constraints on gene expression in flower tissues than in leaves and roots. Moreover, in flowers, the *CO* genetic background clearly dominates over the *CG* background, in striking contrast with the dominance of the *CG* genetic background in the other two tissues. Finally, expression profiles are more conserved between leaves and roots than between flowers and roots.

### Expression similarity and convergence between subgenomes: Flowers differ from roots and leaves

**Fig 7:**
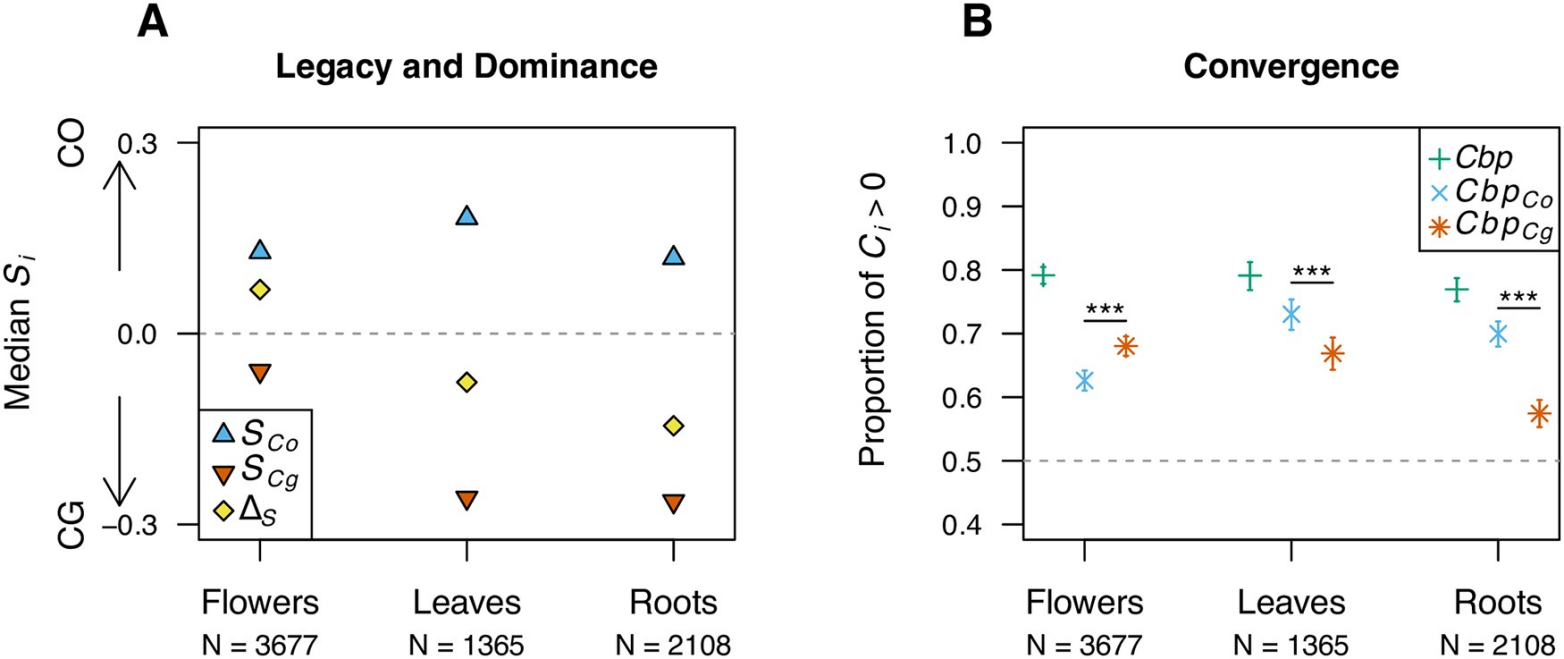
Similarity and convergence indices for differentially expressed genes between subgenomes of *C. bursa-pastoris*. **A**. For each tissue and each subgenome, the median of similarity indices for each subgenome (*S*_*Co*_ and *S*_*Cg*_) are presented as well as the difference between the two indices (Δ_*S*_) that indicates the dominance of one parental genetic background. Grey dotted lines (*S* = 0) indicate level of no bias. **B**. The proportion of transcripts showing convergence (*C*_*i*_ > 0) is reported for the whole genome (green plus signs) or each subgenome (*Cbp*_*Co*_, *Cbp*_*Cg*_). The significance of difference between the subgenome convergence indices is also depicted (binomial test,***, *p* < 0.001). The number of differentially expressed genes considered for each tissue are indicated with N.

To understand better the joint dynamics of expression in the two subgenomes across tissues, and to avoid *a priori* classifications, we defined a new similarity index, *S*, that measures the similarity between mean expression level of each subgenome in each gene and the mean expression level in the parental species for the same gene (see Material and methods § Similarity and Convergence indices). This index is centered on 0, so that *S* < 0 means that the expression of a given transcript from a given subgenome is more similar to the expression of that transcript in *CG*, and *S* > 0 means that its expression is closer to that of *CO*. For all tissues, *S* indices of both subgenomes were biased towards the corresponding parental genome, *i.e*. *Cbp*_*Cg*_ towards *CG* and *Cbp*_*Co*_ towards *CO* (binomial test, all *p* < 0.001). However, the strength of this bias differed between subgenomes and across tissues (Fig 7A). The distributions of *S* values for leaf and root tissues were more spread than the distribution for flowers, meaning that the relative expression in the two subgenomes was globally less constrained in these tissues than in the flower tissue (S4 Fig).

As *S* index reflects the similarity between each subgenome expression and parental expression, the difference between *S* values for a given transcript (Δ_*S*_ = |*S*_*CbpCo*_| − |*S*_*CbpCg*_|) can be viewed as the overall dominance of one parental genetic background over the other (Δ_*S*_ < 0 means dominance of *CG* and Δ_*S*_ > 0 means dominance of *CO*). In flowers, median *S* values for genes that showed significant differential expression between parental species (*FDR* < 0.05) showed dominance of the *CO* over *CG* genetic background (Δ_*S*_ = 0.07), while the opposite pattern—*i.e*. dominance of *CG* back-ground over *CO*—was observed in leaves and roots (Δ_*S*_ = -0.08 and -0.14, respectively; Fig 7A). This pattern was also observed when considering all genes, though it was less pronounced (S4 Fig). Such a dominance cannot only be due to the genes showing strict dominance of one genetic background (*Dominance* category, ∼3-5%), but rather indicate a more global dominance of *trans*-regulation of one genetic background. Indeed, even if *S* indices tended to show a large legacy of parental genome expression, positive correlations between *S*_*Cg*_ and *S*_*Co*_ (Spearman’s *ρ*, all *p* < 0.001) confirmed that both subgenomes were co-regulated in the same direction (S4 Fig), towards *C. orientalis* in flower tissues and towards *C. grandiflora* in leaf and root tissues.

Finally, since subgenomes expression tended to converge, we defined a convergence index to measure the strength of the convergence of each subgenome expression toward the other (*C* index, see Material and methods § Similarity and Convergence indices). Indeed, a closer expression between subgenomes than between parental species can be due to a change in expression of both subgenomes toward an intermediate expression level or to a change in expression of only one subgenome toward the expression level of the other. In all tissues, most convergence indices were positive (Fig 7B and S5 Fig), indicating that the difference in gene expression between subgenomes was generally lower than the difference between parental species; also, the larger the difference in expression between parental species, the stronger the convergence between subgenomes, *C*_*Cbp*_ (Spearman’s *ρ* = 0.63, *ρ* = 0.74, *ρ* = 0.66, respectively for flowers, leaves and roots; all *p* < 0.001). One could expect that the expression patterns of homeologous genes were inherently more correlated because the RNA was extracted from the exact same pool of cells in *C. bursa-pastoris* while it was obviously not the case for the parental species. However, the way the analysis was carried out has likely attenuated this effect. First, the convergence index was computed from the average expression of each subgenome across all *Cbp* accessions, thereby partly breaking such an association. Second, to evaluate the strength of such a potential bias, we also estimated the *C* indices for subgenomes coming either from the same individuals or from different individuals S6 Fig. Although the convergence indices computed from the same individuals were stronger (closer to one) than the ones computed from different individuals, the overall pattern did not change: namely, the vast majority of the convergence indices are positive indicating that the subgenome expression levels converged. Although the overall degree of convergence was the same in the three tissues, the amount of convergence was not the same between the two subgenomes. In flowers, *Cbp*_*Cg*_ expression tended to shift more towards *Cbp*_*Co*_ expression than the converse, while the opposite was true in the two other tissues (Fig 7B). This explains the dominance patterns observed through the *S* indices and confirms the role of unbalanced *trans*-regulation in the present system.

### Genes showing converging expression patterns are enriched for specific functions

Regardless of the tissue considered, the expression profiles did not correspond to specific physical clusters along the genome with transcripts belonging to a given profile being spread across the genome: for each scaffold and each category, the average distance (bp) between two transcripts randomly sampled within a given category was not significantly different than that of two transcripts randomly sampled in different categories (Wilcoxon-Mann-Whitney’s test, all *p* > 0.05, S7 Fig). This suggests that the differential expression is not driven by large-scale epigenetic changes along chromosomes.

Gene ontology analyses revealed that the different expression profile categories (Fig 6) were enriched for different molecular functions (MF, average overlap between categories: 8.9, 9.0 and 8.6% for flowers, leaves and roots tissues, respectively, S6A Table) and biological processes (BP, average overlap, 5.4, 4.2 and 6.0%, S6B Table), though neither MF nor BP of a given category tended to cluster into specific networks. At the tissue level, the different expression profile categories were enriched for different MF and BP with a small average overlap between tissues (MF, 5.3% and BP, 4.8%, S7A and S7B Table), highlighting the specificity of expression regulation in different tissues.

We showed above that the main difference in expression between tissues was in the convergence of the two subgenomes: in flowers, *Cbp*_*Cg*_ expression pattern converged toward that of *Cbp*_*Co*_, while for the two other tissues convergence was in the opposite direction (*Cbp*_*Co*_ toward *Cbp*_*Cg*_). We tested whether the transcripts showing a convergence of *Cbp*_*Cg*_ toward *Cbp*_*Co*_ (hereafter, *Conv*_*Co*_ genes) or a convergence of *Cbp*_*Co*_ toward *Cbp*_*Cg*_ (hereafter, *Conv*_*Cg*_ genes) were enriched for different molecular functions and biological processes. The two gene sets, *Conv*_*Co*_ or *Conv*_*Cg*_ genes, were indeed enriched for GO terms belonging to different clusters (S8 Fig). For instance, in flower tissues, *Conv*_*Co*_ genes are enriched for biological processes involved in the transition between vegetative and reproductive phases, the dormancy of floral meristems and male meiosis, while *Conv*_*Cg*_ genes were enriched for cell redox homeostasis and related biological processes (S8 and S9 Figs). As expected, underlying molecular functions also tended to group into distinct clusters corresponding to different functional networks (S8 and S9 Figs). There was also an enrichment for similar biological processes (e.g., drug transport in flowers, sucrose and carbohydrate metabolisms in leaves and roots) or molecular functions (e.g, RNA, nucleotide and GTP binding or MF related to transporter activity, S8 and S9 Figs) indicating some concerted changes of gene expression between the two subgenomes.

### Deleterious mutations accumulate preferentially on the *C. orientalis* subgenome and are associated with the level of expression

Among the 11 million genomic sites segregating across the five genomes, about 3 million alleles were specific to the *Capsella* species, and 669,675 of these species-specific alleles were annotated for tolerated (*TOL*) and deleterious mutations (*DEL*) by SIFT4G with the *A. thaliana* database, and 432,354 of them were annotated with the *C. rubella* database.

**Fig 8:**
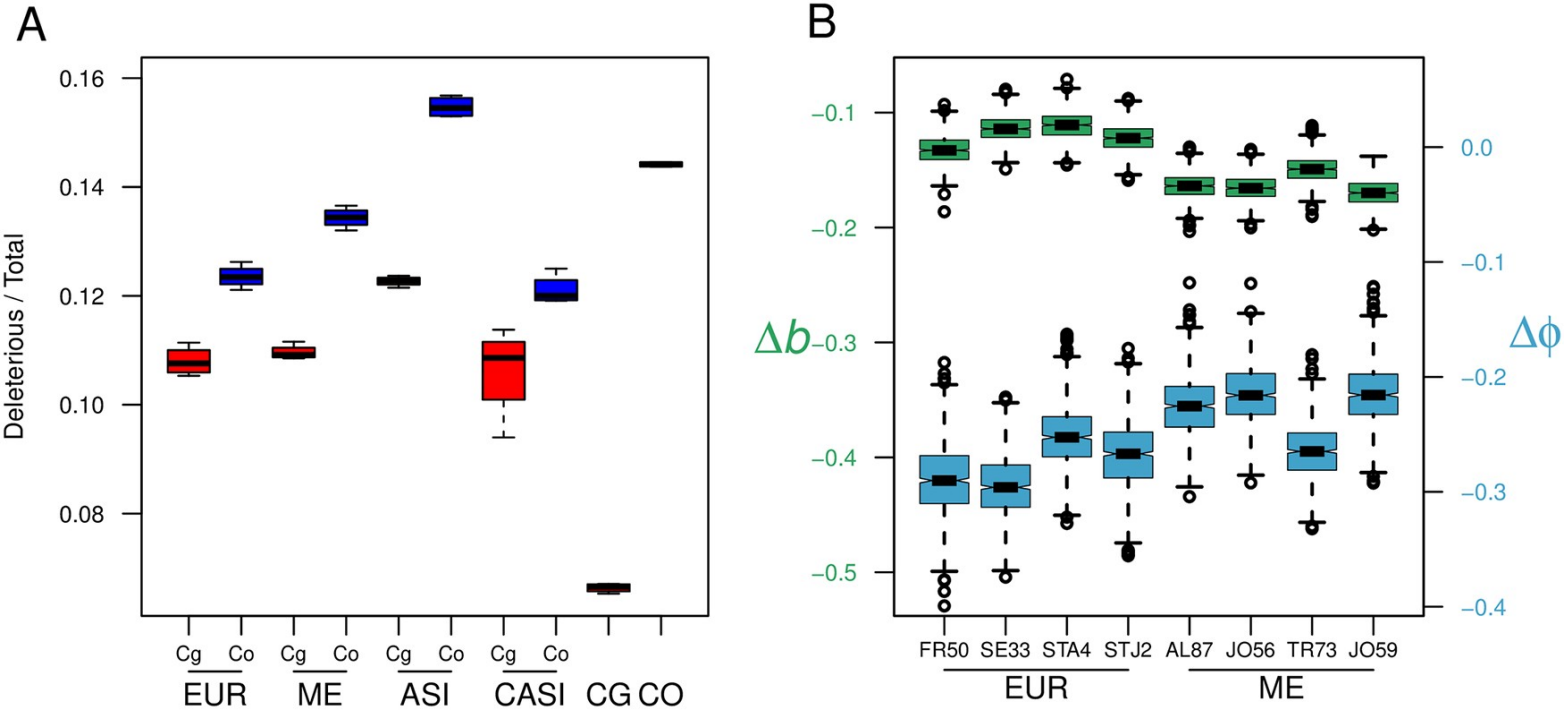
Variation in deleterious mutations in the two subgenomes of *C. bursa-pastoris*. **A**. Proportion of deleterious mutations in the subgenomes and in the parental species. CO, CG, ASI, EUR, ME, CASI correspond to *C. orientalis*, *C. grandiflora*, and four populations of *C. bursa-pastoris*, respectively. The two subgenomes are indicated with Co and Cg. Functional effects were annotated with the *C. rubella* SIFT database (the annotation with *A. thaliana* SIFT database is in the S10 Fig). **B**. Maximum likelihood estimates of parameters of the distribution of deleterious mutations on *Cbp*_*Cg*_ genes. Each box represents the estimates for one accession, with 1000 bootstrap replicates. The estimates are presented as the difference between the estimated parameter for deleterious mutations, *DEL*, and the estimated parameter for synonymous mutations, *SYN* (Δ*b* = *bDEL* − *bSYN*, Δ*φ* = *φDEL* − *φSYN*). Notches represent the median and the 95% confidence interval. The left axis refers to Δ*b* (green boxes), and the right axis refers to Δ*φ* (blue boxes). The estimated parameters (*b* and *φ*) for *DEL* and *SYN* are shown separately in S11 Fig.

The estimated proportion of deleterious mutations among species and among the four populations of *C. bursa-pastoris* were similar independently of whether *A. thaliana* or *C. rubella* was used for SIFT4G annotation (Fig 8A and S10A Fig). Despite a lower number of accessions, the same pattern as in [37] was observed: i) the *C. grandiflora* genome had a lower proportion of deleterious mutations than *C. orientalis* or either subgenome of *C. bursa-pastoris* ii) within *C. bursa-pastoris*, the *Cbp*_*Cg*_ subgenome always had a lower proportion of deleterious mutations than the *Cbp*_*Co*_ subgenome of the same population and iii) among the *C. bursa-pastoris* populations, both subgenomes of the Asian population had a higher proportion of deleterious mutations than the corresponding subgenomes in the other three populations, indicating a higher rate of mutation accumulation in this population. The proportion of deleterious mutations of the newly added CASI population was most similar to that of the EUR population with a larger variance of the proportion of deleterious mutations carried by *Cbp*_*Cg*_ subgenome of CASI accessions (Fig 8A).

We then assessed the distribution of deleterious mutations between the two subgenomes of *C. bursa-pastoris* to test whether they accumulated (i) more in one gene copy than in the other at the homeologue level, as would be expected under a pseudogenization process, (ii) more in one subgenome than in the other as expected if one subgenome predominates (see Material and methods § Difference between species and subgenomes in deleterious mutations). Mutation accumulation pattern between the two subgenomes was thus investigated by estimating the mutation accumulation bias towards *Cbp*_*Cg*_, *b*, and the overdispersion parameter *φ*; a large value of *φ* indicates that mutations tend to accumulate preferentially in one of the two homeologous genes. *b* was positive for synonymous (*SYN*) mutations indicating a mapping bias towards *Cbp*_*Cg*_. *b* was also positive for *DEL* mutations in all accessions (S11 Fig), but much smaller than for *SYN* (*bDEL* < *bSYN*, Fig 8B). This indicates a general bias towards more *DEL* mutations in the *Cbp*_*Co*_ subgenome, despite the mapping bias toward *CG*. The same pattern was observed for *φ* (*φDEL*< *φSYN*, Fig 8B and S11 Fig). Hence, contrary to what is expected under a scenario of pseudogenization, the distribution of deleterious mutations was less over-dispersed than expected at random, suggesting that the accumulation of too many deleterious mutations per gene is prevented; a mechanism that might contribute to the maintenance of both homeologue copies. However, it should be noted that more silenced genes were observed in *Cbp*_*Co*_ than in *Cbp*_*Cg*_ (S12 Fig).

Finally, the relationship between the number of deleterious mutations and the homeologue expressions was investigated by comparing, for each transcript, the difference in number of deleterious mutations ($d_{DEL}=DEL_{Cbp_{Cg}}-DEL_{Cbp_{Co}}$) and the homeologue expression bias ($e=\frac{Cbp_{ij_{Co}}}{Cbp_{ij_{Co}}+Cbp_{ij_{Cg}}}$). The categories where deleterious mutations and expression bias varied in the same direction (*i.e*., *d*_*DEL*_ > 0 and *e* > 0 or *d*_*DEL*_ and *e* < 0) were over-represented (Fisher’s exact test, all *p* < 0.001, S8 Table). This means that the homeologue copy carrying the highest number of deleterious mutations tends to show the lowest expression level. No such association was found when considering transcripts carrying only synonymous mutations (Fisher’s exact test, *p* = 0.57, 0.74 and 0.27 for flowers, leaves and roots tissues, respectively), confirming that the association between deleterious mutations and expression level was not the result of a mapping or annotation bias toward one of the two subgenomes (S8 Table).

## Discussion

The events accompanying the birth of a polyploid species have often been described in rather dramatic terms, with expressions such as “transcriptomic shock” or “massive genome-wide transcriptomic response” often used (*e.g*. [8, 47, 48]). The early and formative years of a young polyploid might indeed be eventful, but what happens afterward may well be less dramatic, especially for tetraploid species with a disomic inheritance such as shepherd’s purse. In the present study, we compared some of the genomic and transcriptomic changes that occurred between *C. bursa-pastoris* and its two parental species *C. grandiflora* and *C. orientalis*. Overall, the emerging picture is one of an orderly and rather conservative transition towards a new “normal” state. A conservative transition, because after around 100,000 generations we can still detect a significant parental legacy effect on both the number of deleterious mutations accumulated and gene expression patterns. And an orderly one too, since the emerging pattern of expression involves a balance between *cis*- and *trans*-regulatory changes suggesting the emergence of coordinated functioning of the two subgenomes. This general impression of a non-stochastic transition process to polyploidy [49] is reinforced by the variation in patterns of gene expression across the three tissues: as one would expect, the expression of both subgenomes in selfing *C. bursa-pastoris* was biased towards the selfing parent *C. orientalis* in flower, whereas in leaf expression of the two subgenomes were mostly similar, and in roots expression was biased towards *C. grandiflora*. This expression bias towards the *C. orientalis* subgenome in flowers despite a higher accumulation of deleterious mutations in this subgenome suggests that the evolution of gene expression is not entirely random.

### Demography and expression: A limited effect of introgression?

Previous studies have stressed the importance of population structure and demographic history in genomic and transcriptomic studies of *C. bursa-pastoris* [36, 37]; [37], for instance, showed a significant admixture between *C. orientalis* and Asian populations of *C. bursa-pastoris*. In the present study, we indeed showed that the overall gene expression pattern reflected the main phylogenetic relationships. Each subgenome was the closest to the parental species from which it was inherited and populations from close geographic areas tended to cluster together, except for Central Asian accessions (CASI), which clustered with European ones even though they were geographically closer to the Asian or Middle-East ones. Most likely these samples were recently introduced to Central Asia, as it was suggested for *C. bursa-pastoris* accessions with European ancestry inhabiting the Russian Far East [35].

When comparing the number of differentially expressed genes between *C. bursa-pastoris* and parental species, no specific trend was detected and Asian accessions were not the closest to *C. orientalis* as one would have expected because of introgression. In leaf and roots tissues, ASI was even closer to *C. grandiflora* than to *C. orientalis*. This can be explained by the fact that the vast majority of the genes (up to 80%) did not show any difference in expression (thus hiding a more subtle signal). Assessing the influence of introgression on expression pattern would require a more thorough investigation, for instance by focusing on genes for which introgression was actually characterized.

### Transition to polyploidy: Compensatory *cis*-*trans* effects, and stabilizing selection

As mentioned above, in the case of a newly formed allopolyploid species one would expect the two homoeologous copies of a gene to be under the influence of *trans*-regulatory elements inherited from both parents and its expression level to first move towards the mean expression of the two parental species. However, different forces could lead to an excess of divergence in subgenome expression compared to what would be expected under a pure drift model. Polyploidy creates a large redundancy in gene function that should free one of the copies from purifying selection. Generally, the copy carrying more deleterious mutations is expected to degenerate, biasing the expression pattern toward one of the two parental species, even if sub- or neo-functionalization can still occur but to a much lower extent. This ought to be particularly true for *C. bursa-pastoris* as one of its parental species, *C. orientalis*, is a selfer that has accumulated more deleterious mutations than the other parent, the outcrossing *C. grandiflora* [37]. This process will be reinforced by the enhancer runways process [27], that should strengthen *cis*-acting elements from the *Cbp*_*Cg*_ subgenome as the *Cbp*_*Cg*_ subgenome has higher heterozygosity and lower genetic load than the *Cbp*_*Co*_ subgenome.

In our study, however, we did not observe any “transcriptomic shock” (as for instance in, [8, 47]) neither major homeologue expression remodeling and/or subgenome expression asymmetry (as in *e.g*. [18]). In contrast, our study, like some others before it [16, 49–52], instead suggests overall conservation of the expression pattern in polyploids and hybrids. And even if a “transcriptomic shock” did take place during the formation of the tetraploid, expression changes have stabilized since then. Some 100,000 years later parental legacy on subgenome expression is still detectable and the two subgenomes’ expression patterns are still closer to each other than that of parental species, clearly indicating that none of the subgenomes has degenerated; as expected, however, the *Cbp*_*Co*_ subgenome carries more silenced genes and a higher proportion of deleterious mutations than *Cbp*_*Cg*_.

Most of the genes were under both *cis-* and *trans-*acting elements; the *No difference* and *Intermediate* expression categories represented up to 70-80% of genes depending on the tissue considered, a percentage similar to that observed in F1 hybrids between *A. thaliana* and *A.arenosa* [52]. Only a small fraction (5 to 10%) of genes showed either almost pure *cis-* (*Legacy* category) or *trans-*regulation (*Dominance* category). While the former can be explained by the absence of crossed *trans*-regulation, the latter could be due to the dominance of transcription factor of one subgenome over the other; though, in both cases, post-hybridization mutations affecting either *cis*- or *trans*-acting elements or both could have evolved. The remaining fraction (up to 15%, *Reverse*, *Compensatory-drift* and *Transgressive*) showed a more complex pattern that is hard to assign to a simple factor but could be in part due to new intertwined *cis-* and *trans*-regulations across subgenomes. It should be noted that such patterns can naturally emerge after hybridization as a byproduct of stabilizing selection on diverging optima [53] for *Transgressive* profiles, on the overall amount of protein produced for *Compensatory-drift* profile, and on the intermediate level of expression for *Reverse* profile, without invoking additional specific processes. To address further this question, it would be interesting to compare auto- and allopolyploids to tease apart the effects of hybridization and genome doubling.

Even though this does not, in any way, alter the conclusion above, we also would like to note here that the classification of overall expression patterns in different categories used in Fig 6 and Table 1 is somewhat arbitrary as some expression patterns are ambiguous and could have been classified in different categories. It should also be pointed out that these classifications were dependent on the chosen False Discovery Rate (FDR). As a control, we reproduced the analysis based on unphased data of *Cbp* expression, with *FDR* < 0.01 and 0.1 (S9 Table). It indicated that the number of genes within the different categories can vary substantially with the different FDR level (mainly because of variation in *No difference* category), however, the main patterns were not altered. Moreover, the main pattern of variation we described was a change in dominance between tissue that is obviously not affected by the bias described before. In part to overcome the limitations inherent to any *a priori* classification, we developed the expression similarity and convergence indices, *S* and *C*, that confirmed our conclusions.

### Level of expression dominance varies across tissues and functions

Allopolyploid species are often examined for unequal expression between homeologous genes because of their hybrid nature but other aspects of gene expression have been less extensively studied. For example, there might be no difference in the relative expression of subgenomes (balanced homeologue expression), but the total amount of transcripts can vary and reflect the dominance of the level of expression of one of the parents [18]. *C. bursa-pastoris* exhibits rather balanced homeologue expression, but the summed expression of the two homeologues shows differentiation across tissues with the dominance of *C. orientalis* expression level in flowers, and *C. grandiflora* level in leaves and roots. The genes with significant expression bias between subgenomes also show strong dominance of *Cbp*_*Co*_ expression over *Cbp*_*Cg*_ in flower. However, a positive correlation between the expression deviation indices of the two subgenomes indicates that this dominance is not primarily caused by up-regulation or down-regulation of one parental copy, but rather unidirectional regulation of homeologous genes as it has been observed, for instance, in cotton and coffee [2, 32, 54]. This convergence could be possible because of the low divergence between the subgenomes of *C. bursa-pastoris* and, hence, the absence of barriers for *trans*-acting regulation of homeologous genes.

An intuitive explanation of this bias in flower tissues could be that this simply reflects the fact that both *C. orientalis* and *C. bursa-pastoris* are selfing species with tiny flowers, in contrast to *C. grandiflora*, an outcrossing species that has large flowers. A way to test this hypothesis would be to compare *C. orientalis* with both *C. grandiflora* and *C. rubella* for the genes implicated in the bias towards *C. orientalis* using root tissues as a control. In contrast, in the non-reproductive leaf and root tissues, expression is biased towards the genome of the outcrossing *C. grandiflora*. Although this interpretation needs further validation, it stands against the genomic shock pattern that implies a disruption of expression patterns.

Finally, although the bias of expression observed between homeologous genes is not strongly shifted towards either subgenome, it is not random either: one subgenome can dominate over the other for a given function or pathway in a given tissue, suggesting constrained evolution in gene expression regulation at a tissue/function level. In many cases, it is not straightforward to explain why a particular subgenome dominates for a particular function, and this could simply be the result of coincidence in neutral evolution of gene regulation networks. In other cases such as flower tissues, however, the observed dominance makes biological sense.

### Both subgenomes of *C. bursa-pastoris* are maintained, but they are not equal

Redundancy of polyploid genomes often assumes evolution of non-functionalization of duplicated genes [55–57] or even of a whole subgenome [38, 44, 58]. When one gene copy of a duplicated gene starts to degenerate, the purifying selection on that copy becomes weaker and the deleterious mutations accumulate further, while the other copy of the gene remains functional and under purifying selection. If non-functionalization is prevalent, deleterious mutations are expected to be more unevenly distributed between the homeologous genes and even between the two subgenomes. We indeed observed more deleterious load in the *Cbp*_*Co*_ subgenome with the absolute load comparison and with the estimated parameter *b* indicating its degeneration. However, the dispersion for deleterious mutations indicated that they tend to be more evenly distributed between the homeologous genes than expected at random. This suggests that *Cbp*_*Co*_ genes cannot degenerate further after a certain amount of genetic load is accumulated. Thus, although the amount of accumulated genetic load differs between subgenomes of *C. bursa-pastoris*, both subgenomes are maintained and there is no large-scale non-functionalization at the gene and subgenome levels.

One might expect the differences between homeologues in accumulation of deleterious mutations would lead to bias in gene expression. For example, *Arabidopsis suecica*, like *C. bursa-pastoris*, is an allopolyploid species with parents characterized by different mating systems: the outcrossing *Arabidopsis arenosa*, and the selfing *Arabidopsis thaliana* [59]. Chang *et al*. [60] observed a bias in expression in favor of the *A. arenosa* subgenome and, among other hypotheses, suggested that this bias could be due to the fact that mildly deleterious alleles are not purged as efficiently from the *A. thaliana* subgenome as from the *A. arenosa* subgenome. In *C. bursa-pastoris*, the *Cbp*_*Co*_ subgenome had a higher proportion of deleterious mutations than the *Cbp*_*Cg*_ subgenome, but there was no strong bias in expression between subgenomes. However, when we paired the amount of derived deleterious mutations with the expression level of each gene and compared homeologous genes, we found that there was a significant association between deleterious mutation bias and expression bias (S8 Table). The homeologous gene with more deleterious mutations tends to have a lower expression level than the other one. Moreover, we also found that there are more silenced genes in *Cbp*_*Co*_, which is the subgenome with a higher proportion of deleterious mutations. These results are in accordance with the hypothesis that the bias in expression is linked to the accumulation of deleterious mutations. Yet, it is worth noting that the expression bias may not necessarily be the result of the biased distribution of deleterious mutations. The homeologue expression bias could also be the cause of the observed deleterious mutation bias, especially considering that we have only investigated the deleterious mutations in coding regions. Purifying selection on the homeologue with lower expression can be weaker [61], therefore it is less efficient in eliminating deleterious mutations. At any rate, the fact that we have a relative dominance of expression of *Cbp*_*Co*_ in flowers and of *Cbp*_*Cg*_ in other tissues, despite *Cbp*_*Co*_ subgenome having a higher proportion of deleterious mutations than *Cbp*_*Cg*_, suggests that parental legacy and functional constraints may also play a major role.

### Conclusion

In 1929, George Shull, one of the most prominent geneticists of his time [62], wrote: “It is considered a matter of fundamental significance that the increase in a number of chromosomes in the *bursa-pastoris* group is correlated with greater variability, greater adaptability, greater vigor, and greater hardiness”. In the present study, the merging of the two parental genomes was not accompanied by major disruptions of the transcriptome. Instead, there was a strong parental legacy and the emergence of a shift in the subgenome expression pattern towards a new “equilibrium” state reflecting the composite nature of the new species. Hence, being a selfer like its *C. orientalis* parent, there was a shift in flower tissues of the expression pattern of the *C. grandiflora* subgenome towards that of *C. orientalis*. Similarly, it seems also possible that the dominance of the *C. grandiflora* inherited subgenome in roots and leaves contributed to the high competitive ability of *C. bursa-pastoris*, which was similar to that of *C. grandiflora* but much higher than that of *C. orientalis* and *C. rubella*, its two self-fertilizing congeners [63, 64]. It therefore seems that the present study, together with those more focused on fitness of *C. bursa-pastoris* [63, 64] contributed to better understanding of the causes of the correlation pointed out almost 100 years ago by Shull.

## Material and methods

### Samples, sequencing and data preparation

We obtained the whole genome and RNA-Seq data from flower, leaf and root tissues of (i) 16 accessions of *C. bursa-pastoris* coming from already characterized populations from Europe (EU), the Middle East (ME) and Eastern Asia (ASI) [35] and from hitherto unstudied Central Asian populations (CASI) and (ii) four accessions each of *C. grandiflora* and *C. orientalis* (Fig 1). The genomic data included both published and newly sequenced genomes (S1 Table). For newly sequenced genomes, DNA was extracted from leaves with the Qiagen DNeasy Plant Mini Kit, libraries were prepared using the TruSeq Nano DNA kit, and 150-bp paired-end reads were sequenced on Illumina HiSeqX platform (SciLife, Stockholm, Sweden). All 72 RNA-Seq libraries (24 accessions × three tissues) were sequenced in this study. For RNA sequencing, seeds were surface-sterilized and germinated as described in [36]. Seedlings were then transplanted into pots (10 × 10 × 10cm) filled with soil seven days after germination and cultivated in one growth chamber (22°C, 16:8h light/dark period, light intensity 150 *μmol*/*m*^2^/*s*). Seven days after the onset of flowering, we collected flower buds, leaves, and roots of visually similar developmental stage. Tissues were snap-frozen in liquid nitrogen, and stored at -80°C before extraction following manufacturer protocol (Plant Total RNA Kit (Spectrum) for flower buds and leaves, and RNeasy Plant Mini Kit (Qiagen) for roots). RNA sequencing libraries were prepared using the TruSeq stranded mRNA library preparation kit including polyA selection and sequenced for 125-bp paired-end reads on Illumina HiSeq 2500 platform (SciLife, Stockholm, Sweden). Sequencing of new samples yielded an average library size of 57 million reads for DNA sequencing and 59 million reads for RNA-Seq.

DNA and RNA-Seq reads were mapped to the *C. rubella* reference genome [65] with Stampy v1.0.22 [66]. To account for the divergence from the reference genome, the substitution rate was set to 0.025 for *C. bursa-pastoris*, 0.02 for *C. grandiflora*, and 0.04 for *C. orientalis*. On average, 85%, 90% and 85% of the DNA reads were successfully mapped for the corresponding three species and 98% in all species for RNA mapping. This yielded an average coverage of 51x and 52x for DNA and RNA data, respectively. Genotyping of DNA and RNA-Seq alignments were performed using HaplotypeCaller from the Genome Analysis Tool Kit (GATK) v3.5 [67] as described in [37]. The subgenomes of *C. bursa-pastoris* were phased with HapCUT version 0.7 [68] following the procedure by [37]. The quality of this phasing procedure was ascertained by comparing the phased subgenomes with the subgenome assembly obtained by [45]. The unphased expression data was generated for non-overlapping feature positions (option: *-m union*) using the *htseq-count* program from HTSeq v0.6.1 [69]. To compare the expression between the two subgenomes of *C. bursa-pastoris*, homeologue-specific counting of alleles was performed using *ASEReadCounter* from GATK and phased according to the phased genomic data. We analyzed only the counts of SNPs that showed no strong deviation from the 0.5 mapping ratio in DNA data defined with a statistical model developed by [70]. To correct for potential bias in homeologue count data due to the uneven density of SNPs and/or uneven coverage along the gene, we scaled the homeologue expression counts using the unphased data and the allelic ratio from the phased data.

### Population structure

Principal component analyses were performed using the *ade4* R package [71]. A neighbor-joining phylogenetic tree was reconstructed from the absolute genetic distance in genomic SNPs with the *ape* R package [72]. A hierarchical distance clustering with bootstrap support was perfromed in the *pvclust* R package, [73].

### Gene expression analyses

Differential gene expression analyses were carried out in *edgeR* [74]. The TMM normalization for different library sizes [74] was used for differential gene expression analyses, while for all other analyses, we used the count per million (CPM) normalization (one was added to every gene count to bypass log-transformation of zero expression). Phased counts were normalized by the mean library size of the two subgenomes $\left(\frac{Cbp_{Co}+Cbp_{Cg}}{2}\right)$ and only genes showing no strong mapping bias were retained (see below). For both datasets (unphased or phased), only genes with at least one sample having a non-zero expression in every population/species were kept.

Differences between the two subgenomes (homeologue-specific expression) were assessed with the integration of the information from both RNA and DNA data to exclude highly biased SNPs and to account for the noise in read counts due to statistical variability. The data were analyzed using the three-stage hierarchical Bayesian model for allelic read counts developed by [70]. The model was implemented using Markov chain Monte Carlo (MCMC) with 200,000 iterations with burn-in of 20,000 and thinning interval of 100. Each analysis was run three times to assess convergence. The significance of homeologue-specific expression (HSE) was defined from a Bayesian analog of the false discovery rate (*FDR* < 0.05).

Expression patterns in *C. bursa-pastoris* and its parental species were classified into categories based on significant and non-significant differential expression defined with *edgeR* [74]. We considered the four genomes/subgenomes (*CG*, *CO*, *Cbp*_*cg*_, and *Cbp*_*co*_) and three possibilities for each of the six pairwise comparisons (significantly over, under or equally expressed, *FDR* < 0.05), and grouped the resulting combinations into seven main categories: *No difference*, *Intermediate*, *Legacy*, *Reverse*, *Dominance*, *Compensatory drift*, and *Transgressive* (see the Results for categories description). We also performed similar analysis for the unphased total *C. bursa-pastoris* expression (thus considering only three pairwise comparisons) by classifying the expression patterns into four major categories: 1) *no differential expression*, when no significant differences are detected in any of the three pairwise comparisons, 2) *intermediate*, when the expression of *C. bursa-pastoris* (*Cbp*) is intermediate between *C. grandiflora* (*CG*) and *C. orientalis* (*CO*), 3) *dominance* of one of the parents over the other, when the mean expression of *C. bursa-pastoris* is equal to only one parental species and the two parents are significantly different, and finally 4) *transgressive*, when the mean expression of *C. bursa-pastoris* is outside the range of expression of both parents and statistically significantly different from the parental species with the closer level of expression.

### Similarity and convergence indices

To quantify the similarity between each subgenome expression level and the expression level in the parental species, we developed a similarity index (*S*). For each transcript *i* and each subgenome *j* ∈ {*Cbp*_*Cg*_, *Cbp*_*Co*_}, *S* was computed as the subgenome relative expression deviation from the mean expression level in the parental species, $\mu_{i}=\left(E_{i_{CO}}+E_{i_{CG}}\right)/2$:

$$\begin{array}{c} S_{ij}=\frac{E_{ij}-\mu_{i}}{\mu_{i}}, \end{array}$$

Where (*E*_*ij*_) is the average expression of a given transcript *i* in a given genetic background *j* (*CO* or *CG* for parental species, and *Cbp*_*Cg*_ or *Cbp*_*Co*_ for subgenomes of *C. bursa-pastoris*). This index is centered on 0 and oriented (i.e, $S_{ij}=\frac{E_{ij}-\mu_{i}}{\mu_{i}}\times -1$ when $E_{i_{CG}}>E_{i_{CO}}$), so that if *S*_*ij*_ < 0 or *S*_*ij*_ > 0, the expression of a given transcript in a given subgenome is more similar to the expression of that transcript in *CG* or *CO*, respectively. The difference between the absolute values of the indices values for *Cbp*_*Cg*_ and *Cbp*_*Co*_, $\Delta_{S_{i}}=|S_{iCbpCo}\left|-\right|S_{iCbpCg}|$ was used as a measure of dominance of one of the parental genetic background.

Finally, for each gene that was differentially expressed between the two parental species, a convergence index, *C*, was computed from the absolute difference in expression for:

- subgenomes: Δ_*sub*_ = |*Ei*_*Cg*_ − *Ei*_*Co*_|
- parental species: Δ_*par*_ = |*Ei*_*CG*_ − *Ei*_*CO*_|
- each subgenome and the *opposite* parental species: Δ_*Cg*_ = |*Ei*_*Cg*_ − *Ei*_*CO*_| and Δ_*Co*_ = |*Ei*_*Co*_ − *Ei*_*CG*_|.

These differences correspond to the phylogenetic distances (Fig 1A). In principle, if the regulation of gene expression in *Cbp*_*Cg*_ is independent of the regulation of gene expression in *Cbp*_*Co*_, then the overall Δ_*sub*_, Δ_*par*_, Δ_*Cg*_ and Δ_*Co*_ are expected to be equal. To compare these quantities, for each transcript *i*, we used a convergence index (*C*_*i*_):

$$\begin{array}{c} C_{i}=\frac{\Delta_{par}-\Delta_{x}}{max(\Delta_{par},\Delta_{x})}, \end{array}$$

So, $C_{Cbp_{Cg}}$ measures the expression convergence of *Cbp*_*Cg*_ toward *Cbp*_*Co*_, $C_{Cbp_{Co}}$ measures the expression convergence of *Cbp*_*Co*_ toward *Cbp*_*Cg*_, and *C*_*Cbp*_ measures the overall subgenomes convergence within *Cbp*. Δ_*x*_ stands for either Δ_*Co*_, Δ_*Cg*_ or Δ_*sub*_, respectively. *C*_*i*_ thus ranges from -1 to 1, with positive values indicating more similar expression between the subgenomes of *C. bursa-pastoris* than between parental species, and negative values indicating increased differences between subgenomes; the closer *C*_*i*_ to 0, the more similar are the expression patterns to parental species.

### Gene ontology enrichment test

Gene ontology (GO) enrichment tests were performed using the *topGO* R package [75]. The GO term annotation was downloaded from PlantRegMap (http://plantregmap.cbi.pku.edu.cn) and we used a custom background list of genes that included only the expressed genes for which phasing was possible in the relevant tissue. Fisher’s exact-test procedure (*weight* algorithm) was performed to assess the enrichment (*p* < 0.05) for either molecular functions (MF) or biological processes (BP). Finally, the *REViGO* software [76] was used to remove GO terms redundancy and to cluster remaining terms in a two-dimensional space derived by applying multidimensional scaling to a matrix of the GO terms semantic similarities. Cytoscape v3.6.1 was used to visualize GO terms networks [77].

### Difference between species and subgenomes in deleterious mutations

Mutations were classified into tolerated and deleterious (DEL) using SIFT4G [78]. We used *C. rubella* [37] and *Arabidopsis thaliana* (TAIR10.22) SIFT4G reference databases. This helps avoid reference bias towards *C.rubella* away from calling mutations to be deleterious in the *C. grandiflora* homeologue. We considered only the mutations that accumulated after speciation of *C. bursa-pastoris* and identified mutations specific to *C. grandiflora*, *C. orientalis*, the two subgenomes of *C. bursa-pastoris*, and *Neslia paniculata* that was used as an outgroup here. All estimates were relative to the total number of SIFT4G annotated sites to minimize the bias associated with variation in missing data as in [37]. Only the European and Middle Eastern populations were used in further analysis of the distribution of deleterious mutations, in order to exclude the effect of gene flow between *C. orientalis* and the Asian population of *C. bursa-pastoris* [37].

We assessed the distribution of deleterious mutations between the two subgenomes of *C. bursa-pastoris* to test whether they accumulated (i) more in one gene copy than in the other at the homeologue level, as would be expected under a pseudogenization process, (ii) more in one subgenome than in the other as expected if one subgenome predominates. Under the null hypothesis (random accumulation without subgenome bias) the distribution of deleterious mutations between the two subgenomes should follow a binomial distribution with mean 1/2. Under the first hypothesis, the distribution should be more dispersed with the same mean, which can be modeled by a Beta-binomial distribution. Under the second hypothesis, the mean should differ from 1/2. However, over-dispersion and bias can also occur because of missing data and sampling error, we thus used synonymous mutations (SYN) to control for this and built the correct null distribution. To do so, we developed a maximum likelihood method implemented in *R* [79] as follows. First, we identified a most likely probability distribution model by fitting four models to the SYN dataset, where *nSYN* is the sum of *SYN* mutations occurring on both homeologous genes and *kSYN* is the number of *SYN* mutations occurring on *Cbp*_*Cg*_ genes. The four models are:

- M1: *kSYN* ∼ *B*(*nSYN*, 0.5), a binomial distribution with no bias between *Cbp*_*Cg*_ and *Cbp*_*Co*_,
- M2: *kSYN* ∼ *B*(*nSYN*, 0.5 + *b*), a binomial distribution with bias,
- M3: *kSYN* ∼ *BB*(*nSYN*, 0, *φ*), a beta-binomial distribution with no bias,
- M4: *kSYN* ∼ *BB*(*nSYN*, *b*, *φ*), a beta-binomial distribution with bias.

For convenience, the beta-binomial distribution:

$$\begin{array}{c} k∼BB(n,\alpha ,\beta ) \end{array}$$

was re-parameterized as:

$$\begin{array}{c} k∼BB(n,b,\varphi ), \end{array}$$

where $b=\frac{\alpha }{\alpha +\beta }-0.5$ and $\varphi =\frac{1}{\alpha +\beta }$ [80, 81]. In this way, the parameter *b* was a measure of the bias towards the *Cbp*_*Cg*_ genes, and *φ* was a measure of the variance of the probability that a mutation is found within the *Cbp*_*Cg*_ homeologues, and can be interpreted as an index of overdispersion. A large value of *φ* indicates that mutations tend to accumulate preferentially in one of the two homeologous genes, and a small value of *φ* indicates that mutations are more evenly distributed between them. We calculated the likelihood of each model and chose the best-fitting model with a hierarchical likelihood ratio test (hLRTs). After choosing the beta-binomial distribution with bias as the most likely null distribution, we estimated the parameters *b* and *φ*. We introduced a new set of models to test for the specific features of the distribution of deleterious mutations:

$$\begin{array}{l} kSYN\sim BB(nSYN,bSYN,\varphi SYN),\\ kDEL\sim BB(nDEL,bSYN,\varphi SYN). \end{array}$$

The null model assumes that both parameters *b* and *φ* are the same for the *SYN* and *DEL* datasets, while the alternative models allow the *DEL* dataset to have different parameters from the *SYN* dataset: only *bDEL*, only *φDEL*, or both *bDEL* and *φDEL* were allowed to vary. We calculated the likelihood of each model, chose the best fitting model with hierarchical likelihood ratio tests (hLRTs) and estimated the parameters of the selected model. Bootstrap estimates of confidence intervals were estimated with 1000 bootstrap replicates.

### Relationship between deleterious mutations and gene expression

The SIFT4G annotation of the *C. rubella* database was used to match the gene IDs of the mutation and expression data. For each tissue, the relationship between the bias in the number of deleterious mutations between subgenomes and the bias in homeologue expression was investigated by calculating, for gene *i* in accession *j*, the difference (*d*_*ij*_) in the number of deleterious mutations (*DEL*) between homeologous gene pairs:

$$\begin{array}{c} d_{ij}=DEL_{ij_{Cg}}-DEL_{ij_{Co}}. \end{array}$$

The expression ratio between the homeologues of genes with significant HSE was used as a measure of homeologue expression bias:

$$\begin{array}{c} e_{ij}=\frac{Cbp_{ij_{Co}}}{Cbp_{ij_{Co}}+Cbp_{ij_{Cg}}} \end{array}$$

Genes were further classified into four categories according to the deleterious mutations bias, *d*, and homeologue expression bias, *e*:

1. *d* > 0 and *e* > 0.5;
2. *d* > 0 and *e* < 0.5;
3. *d* < 0 and *e* > 0.5;
4. *d* < 0 and *e* < 0.5.

Genes with no bias in the distribution of deleterious mutation (*d* = 0) or no significant HSE (*FDR* < 0.05) were removed from the analysis. Fisher’s exact test was then used to test for independence between the difference in the number of deleterious mutations (*d*) and homeologue expression bias (*e*). As a control, the whole analysis was reproduced with *d*_*ij*_ computed from the number of synonymous mutations in genes with no *DEL* mutations. In addition, we also compared the number of silenced genes (genes with zero expression values) of each subgenome of *C. bursa-pastoris*, to check if there was a relationship between genetic load and silenced genes.

### Data access

SRA numbers of the previously published samples are listed in S1 Table. New sequences of the DNA and RNA samples are avaliable under the project PRJNA533007 at NCBI and their SRA numbers are also provided in S1 Table. Phased and unphased genomic and expression data are deposited to the Open Science Framework Repository (DOI 10.17605/OSF.IO/G6H57) [82].

## Supporting information

S1 Fig Neighbor-joining tree of the genomic data of three *Capsella* species. (PDF)

S2 Fig Distance clustering dendrogram of gene expression data for different populations. (PDF)

S3 Fig Distance clustering dendrogram of gene expression data of separate samples. (PDF)

S4 Fig Distribution of the similarity index for each subgenome of *C. bursa-pastoris*. (PDF)

S5 Fig Subgenomes convergence in *C. bursa-pastoris* given differentiation from parental expression. (PDF)

S6 Fig Convergence indices estimated for *C. bursa-pastoris* subgenomes coming from the same and from different individuals. (PDF)

S7 Fig Expression profile regarding genome position. (PDF)

S8 Fig Two-dimensional semantic space representation of significantly enriched GO categories in genes showing convergence in expression. (PDF)

S9 Fig Network shared names of enriched biological processes (A, B and C) or molecular functions (D, E and F) GO term for genes showing convergence in expression between subgenomes in flowers (A and D), leaves (B and E) or roots tissues (C and F). (PDF)

S10 Fig Proportion of deleterious mutations in the two subgenomes of *C. bursa-pastoris* and the genomes of its parental species. (PDF)

S11 Fig Maximum likelihood estimated parameters of the distribution of deleterious mutations on *Cbp*_*Cg*_ genes. (PDF)

S12 Fig The difference in the number of silenced genes between subgenomes of *C. bursa-pastoris*. (PDF)

S1 Table Samples information. (PDF)

S2 Table Differential gene expression between three *Capsella* species in three tissues. (PDF)

S3 Table Differential gene expression between *Capsella* species/population in three tissues. (PDF)

S4 Table Expression ratio between the two subgenomes of *C. bursa-pastoris* across populations in three tissues. (PDF)

S5 Table Differentially expressed genes between tissues (A) and populations within tissues (B) for each *C. bursa-pastoris* subgenomes. (PDF)

S6 Table Overlap between expression profiles in gene ontology term enrichment for biological processes (A) and molecular functions (B). (PDF)

S7 Table Overlap between tissue in expression profiles gene ontology term enrichment for biological processes (A) and molecular functions (B). (PDF)

S8 Table Contingency table of number of genes per category based on deleterious mutation and homelogue expression bias. (PDF)

S9 Table Gene expression levels in *C. bursa-pastoris* and its parental species with different FDR thresholds. (PDF)
