# Supplementary material for: Towards the new normal: Transcriptomic convergence and genomic legacy of the two subgenomes of an allopolyploid weed (Capsella bursa-pastoris)
Source: PLoS Genet. 2019 May 13;15(5):e1008131. doi: 10.1371/journal.pgen.1008131 (PMC6532933; doi:10.1371/journal.pgen.1008131)
Supplement: S1 Fig — (PDF) [file pgen.1008131.s001.pdf]

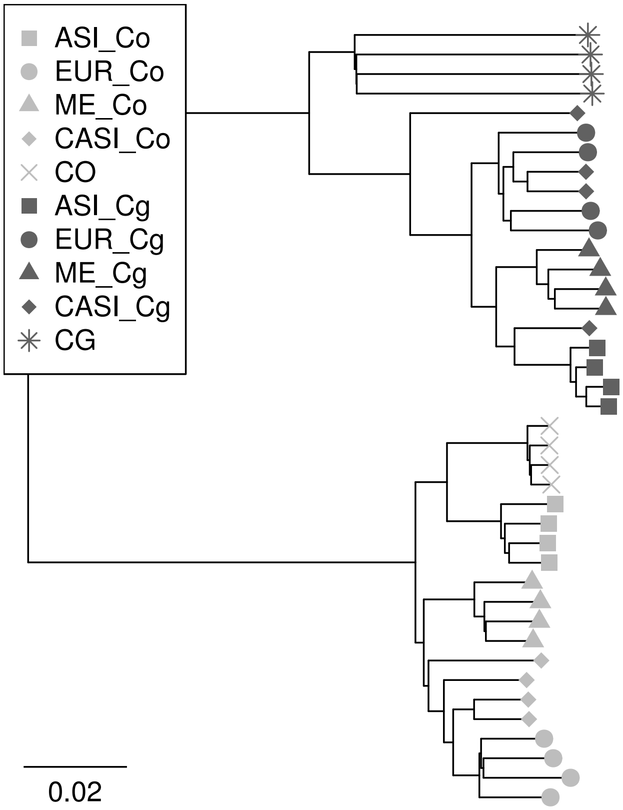

Fig. S1. Neighbor-joining tree of the genomic data of three *Capsella* species. CO, CG, ASI, EUR, ME, CASI correspond to textitC. orientalis, *C. grandiflora*, and four populations of *C. bursa-pastoris*, respectively. The two subgenomes are indicated with Co and Cg. The tree was reconstructed from 11Mb of SNPs and the distance was then scaled to whole genome variation.
