# Supplementary material for: Towards the new normal: Transcriptomic convergence and genomic legacy of the two subgenomes of an allopolyploid weed (Capsella bursa-pastoris)
Source: PLoS Genet. 2019 May 13;15(5):e1008131. doi: 10.1371/journal.pgen.1008131 (PMC6532933; doi:10.1371/journal.pgen.1008131)
Supplement: S2 Fig — (PDF) [file pgen.1008131.s002.pdf]

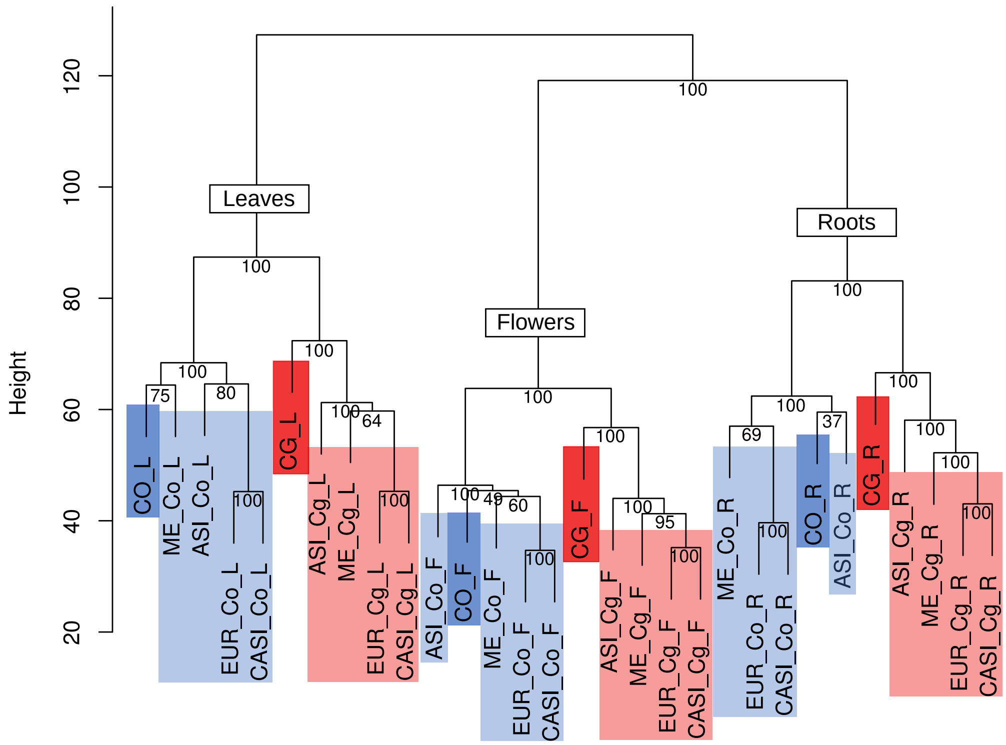

Fig. S2. Distance clustering dendrogram of gene expression data for different populations. Clustering was performed using Euclidean distances and the average agglomerative method on mean expression values for each population (10,403 genes). CO and CG correspond to diploid species *C. orientalis* and *C. grandiflora*, respectively. The Asian, European and Middle Eastern populations of *C. bursa-pastoris* are called ASI, EUR and ME, and the two subgenomes are indicated with Co and Cg. F, L, and R stand for flower, leave and root tissues, respectively. Bootstrap support was generated from 1000 replicates. Color boxes indicate different species and subgenomes.
