# Supplementary material for: Towards the new normal: Transcriptomic convergence and genomic legacy of the two subgenomes of an allopolyploid weed (Capsella bursa-pastoris)
Source: PLoS Genet. 2019 May 13;15(5):e1008131. doi: 10.1371/journal.pgen.1008131 (PMC6532933; doi:10.1371/journal.pgen.1008131)
Supplement: S3 Fig — (PDF) [file pgen.1008131.s003.pdf]

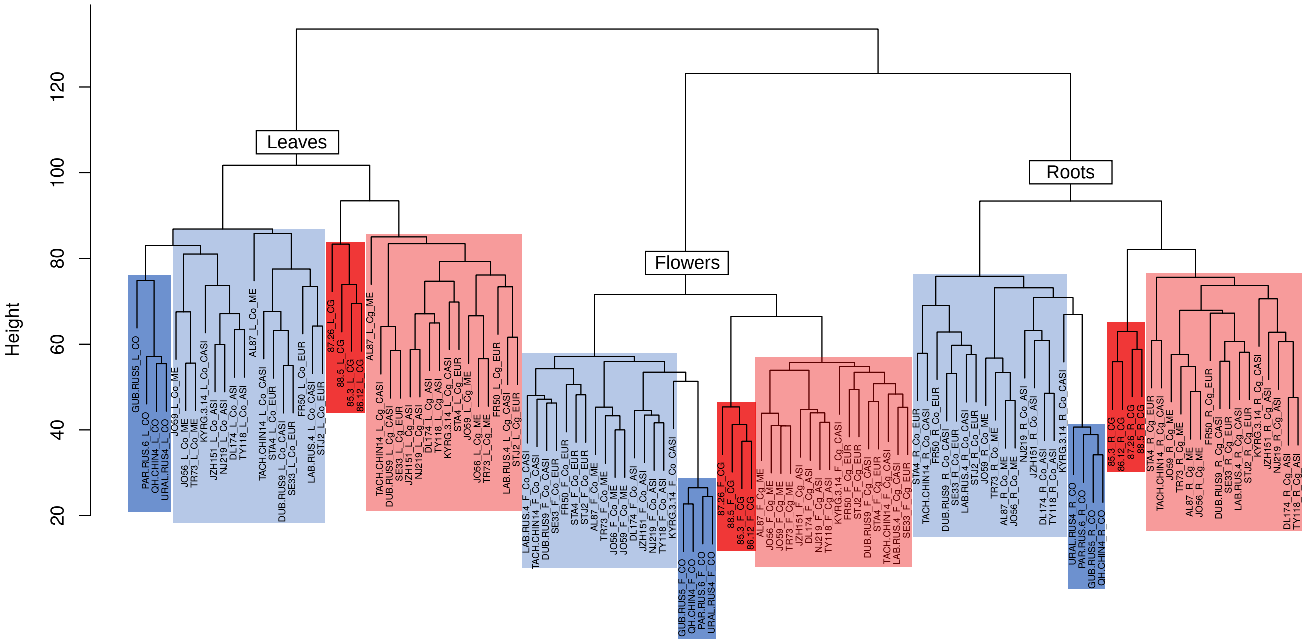

Fig. S3. Distance clustering dendrogram of gene expression data of separate samples. Clustering was performed using Euclidean distances and the average agglomerative method on 11959 genes. Each label indicates an accession number, tissue, and population. In the labels, CO and CG correspond to diploid species *C. orientalis* and *C. grandiflora*, respectively. The Asian, Central Asian, European and Middle Eastern populations of *C. bursa-pastoris* are called ASI, CASI, EUR and ME, and the two subgenomes are indicated with Co and Cg. F, L, and R stand for flower, leave and root tissues, respectively. Color boxes indicate different species and subgenomes.
