# Supplementary material for: Towards the new normal: Transcriptomic convergence and genomic legacy of the two subgenomes of an allopolyploid weed (Capsella bursa-pastoris)
Source: PLoS Genet. 2019 May 13;15(5):e1008131. doi: 10.1371/journal.pgen.1008131 (PMC6532933; doi:10.1371/journal.pgen.1008131)
Supplement: S4 Fig — (PDF) [file pgen.1008131.s004.pdf]

Differentially expressed genes

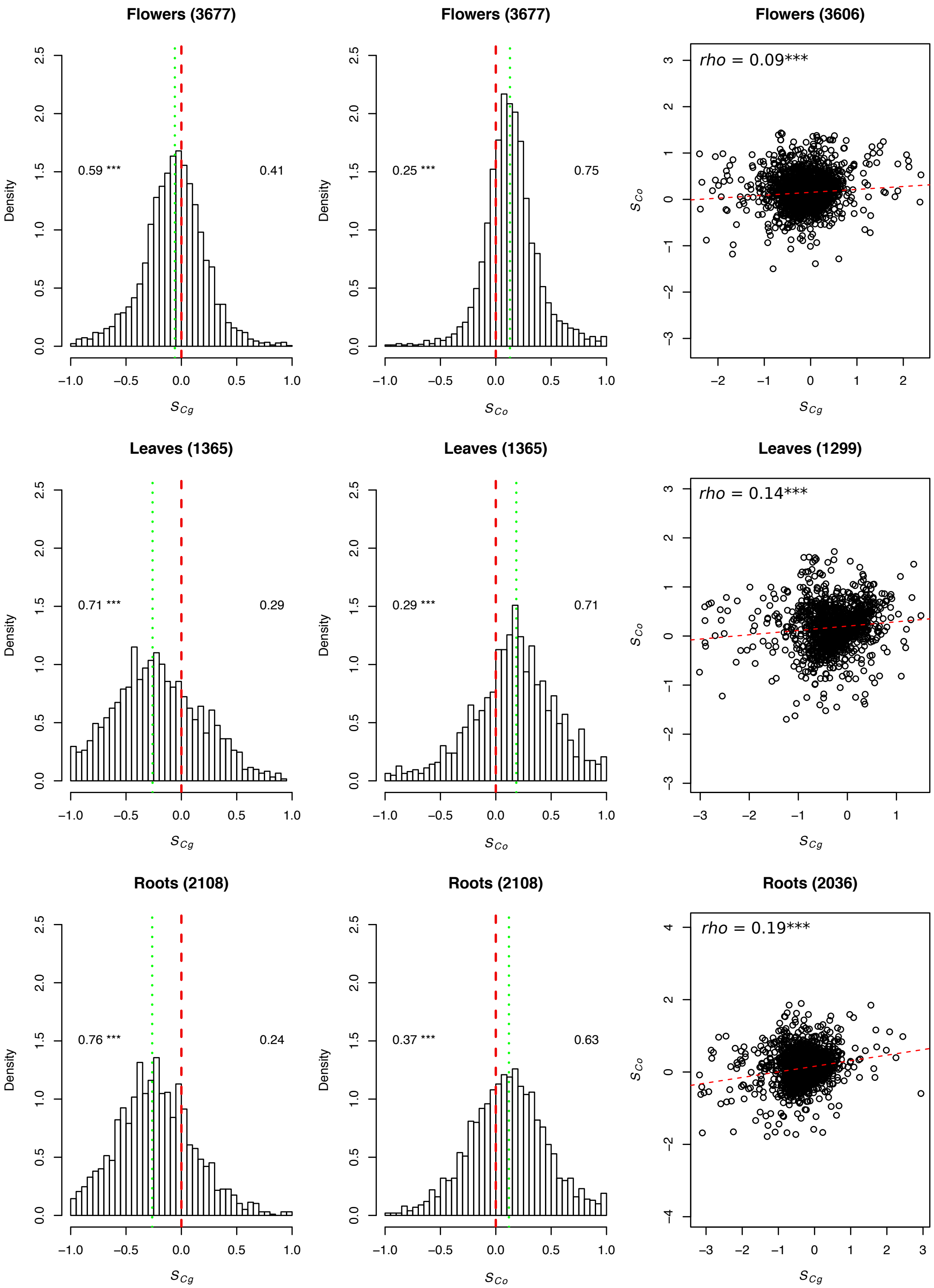

Continue on the next page

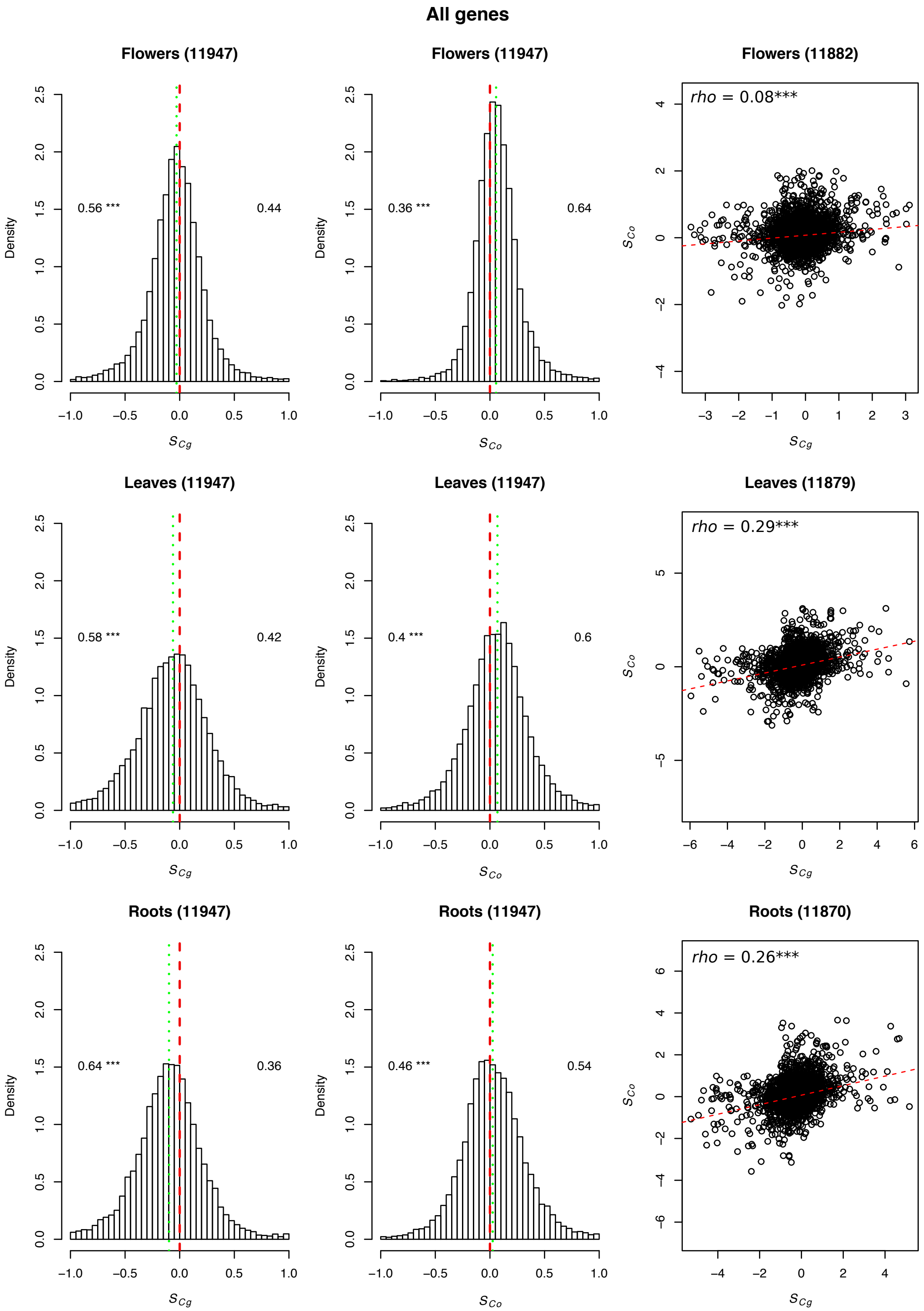

Fig. S4. Distribution of the similarity index for each subgenome of *C. bursa-pastoris*. The similarity index was computed for each subgenome (Cg, left panels and Co, right panels) and for each tissue separately (flowers, top panels, leaves, middle panels and roots bottom panels). The number of genes considered in each analysis is indicated within parentheses. S values < 0 means bias towards CG while S values > 0 bias toward CO. The red dashed line represents S = 0 and the green dotted line is the median S-value. Numbers are proportions of genes bias toward each parental genome. Note that the x-axis is truncated to the interval [-1,1]. Right panels are correlations between S_{Co} and S_{Cg}, red dashed lines are linear regressions between both factors, Spearman's correlation coefficients (ρ) are indicated as well as their significance (***, $p < 0.001$).
