# Supplementary material for: Towards the new normal: Transcriptomic convergence and genomic legacy of the two subgenomes of an allopolyploid weed (Capsella bursa-pastoris)
Source: PLoS Genet. 2019 May 13;15(5):e1008131. doi: 10.1371/journal.pgen.1008131 (PMC6532933; doi:10.1371/journal.pgen.1008131)
Supplement: S5 Fig — (PDF) [file pgen.1008131.s005.pdf]

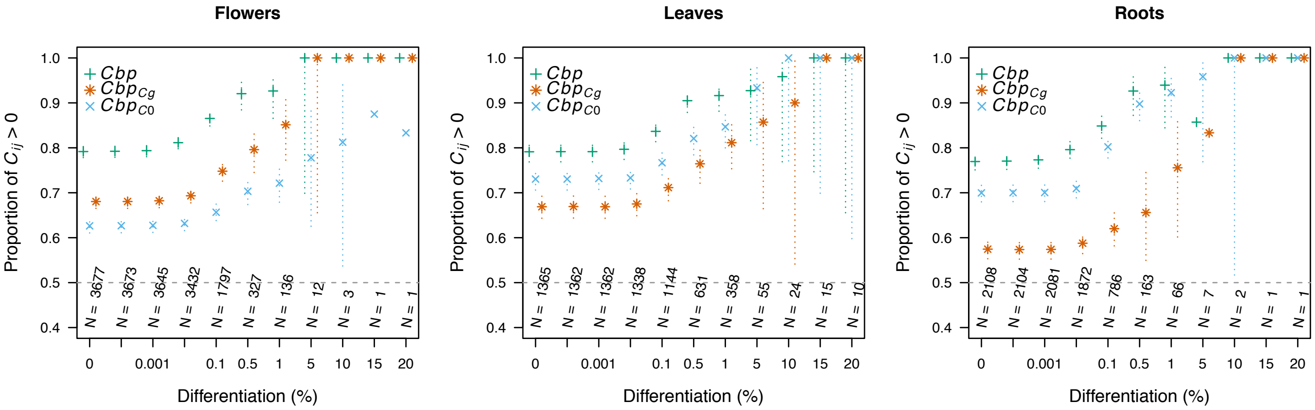

Fig. S5. Subgenomes convergence in *C. bursa-pastoris* given differentiation from parental expression. For each tissue, and each subgenome i, the proportion of transcripts j showing convergence (*i.e.*, $C_{ij} > 0$) are reported as a function of differentiation from parental expression with the associated 95% confidence intervals (dotted lines, non-significant when absent). As an indication of power for binomial tests, the number of gene considered for the various threshold of differentiation for the overall Cbp convergence are indicated (N).
