# Supplementary material for: Towards the new normal: Transcriptomic convergence and genomic legacy of the two subgenomes of an allopolyploid weed (Capsella bursa-pastoris)
Source: PLoS Genet. 2019 May 13;15(5):e1008131. doi: 10.1371/journal.pgen.1008131 (PMC6532933; doi:10.1371/journal.pgen.1008131)
Supplement: S6 Fig — (PDF) [file pgen.1008131.s006.pdf]

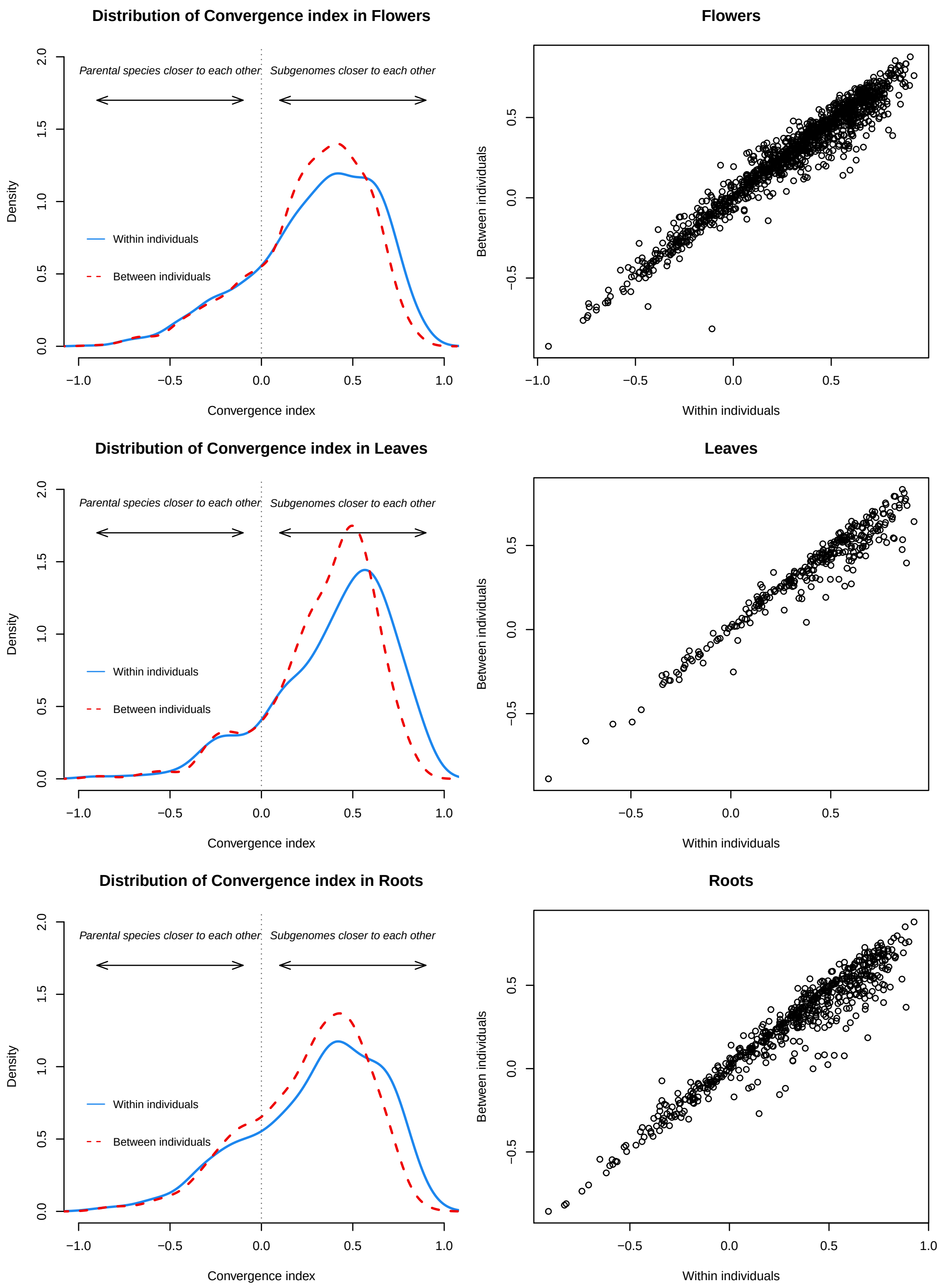

Fig. S6. Overall convergence indices estimated for *C. bursa-pastoris* subgenomes coming from the same individuals or from different individuals. The overall convergence indices, C, were computed from Cbp_{Co} and Cbp_{Cg} subgenomes expression levels from the same individuals (within individuals) or from different individuals (between individuals). The left panels show the distribution of C indices, and the right panels show the correlation between the two groups of estimates.
