# Supplementary material for: Towards the new normal: Transcriptomic convergence and genomic legacy of the two subgenomes of an allopolyploid weed (Capsella bursa-pastoris)
Source: PLoS Genet. 2019 May 13;15(5):e1008131. doi: 10.1371/journal.pgen.1008131 (PMC6532933; doi:10.1371/journal.pgen.1008131)
Supplement: S7 Fig — (PDF) [file pgen.1008131.s007.pdf]

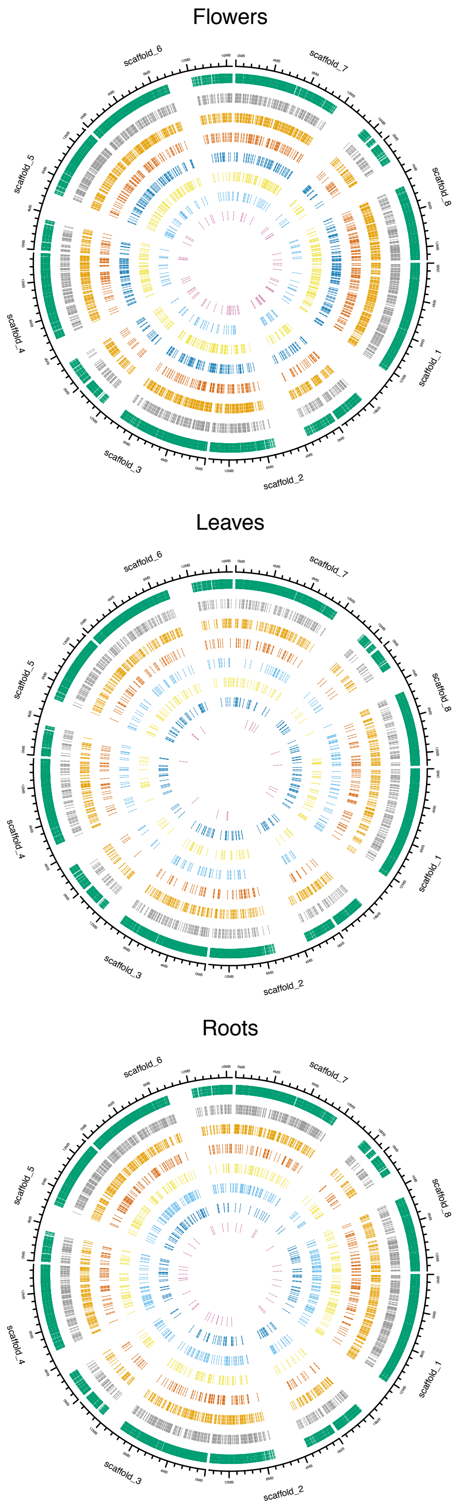

Fig. S7. Expression profile regarding genome position. For each tissue, each transcript is positioned on a scaffold by a vertical bar (given $C.\ rubella$ annotation). Concentric circles and colors correspond to the different expression profiles: *No differences*, green; *Transgressive*, grey; *Intermediate*, orange; *Legacy*, red; *Compensatory drift*, yellow; *Dominance*, blue (Cbp_{Co} dark and Cbp_{Cg} ligth); *Reverse*, pink. Profiles are organized given their importance (number of transcript).
