# Supplementary material for: Towards the new normal: Transcriptomic convergence and genomic legacy of the two subgenomes of an allopolyploid weed (Capsella bursa-pastoris)
Source: PLoS Genet. 2019 May 13;15(5):e1008131. doi: 10.1371/journal.pgen.1008131 (PMC6532933; doi:10.1371/journal.pgen.1008131)
Supplement: S8 Fig — (PDF) [file pgen.1008131.s008.pdf]

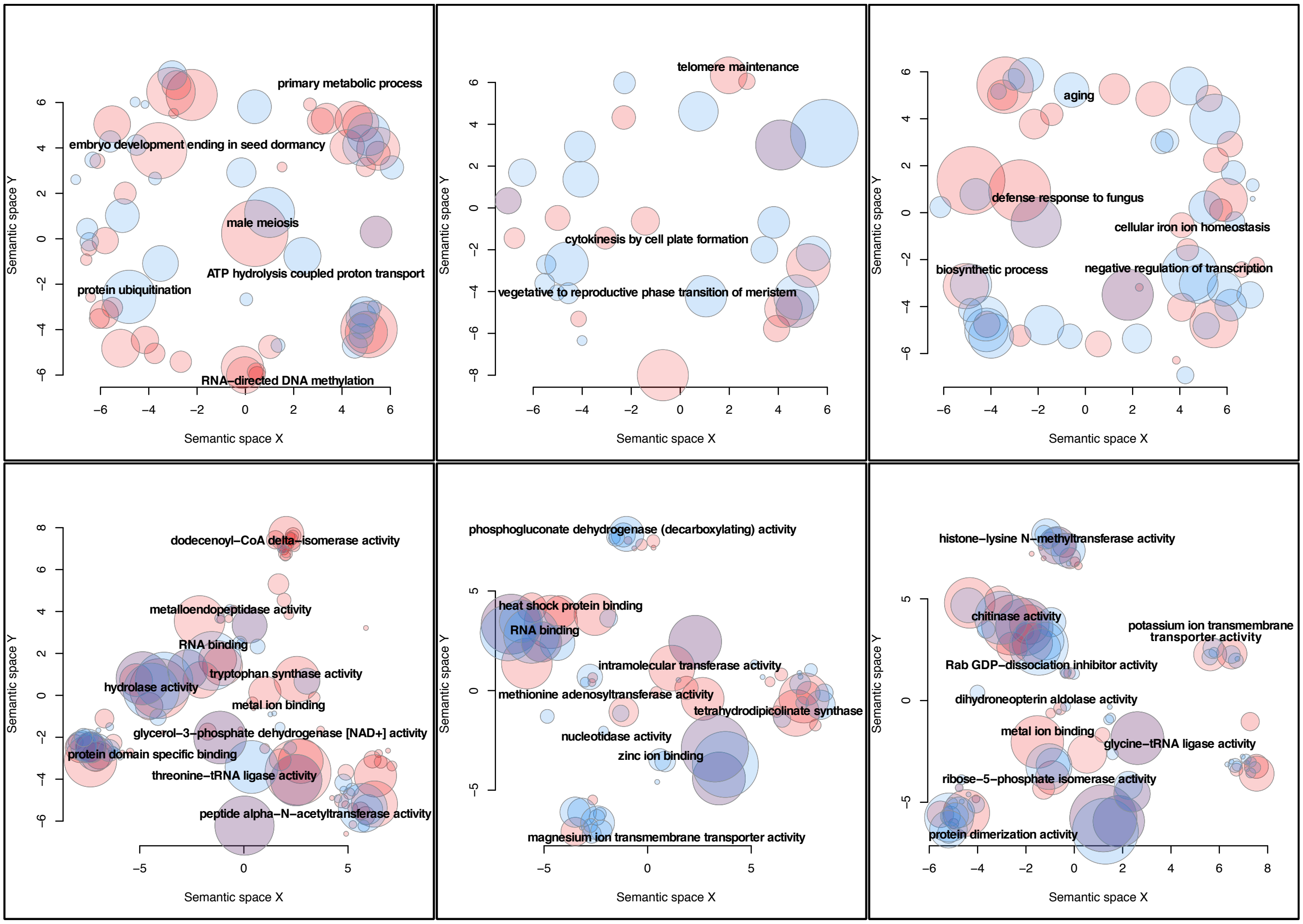

Fig. S8. Two-dimensional semantic space representation of significantly enriched GO categories in genes showing convergence in expression. Multidimensional scaling to a matrix of the GO terms' semantic similarities (see text for details). Semantic representation of GO categories is colored according to the significant over-representation ($p < 0.05$) in genes showing a convergence of expression either of Cbp_{Cg} toward Cbp_{Co} (red) or of Cbp_{Co} toward Cbp_{Cg} (blue) for biological processes (top panels) or molecular functions (bottom panels). The circle diameter is proportional to the number of aggregated GO terms. Description of the GO term with the highest dispensability in the group is reported.
