# Supplementary material for: Towards the new normal: Transcriptomic convergence and genomic legacy of the two subgenomes of an allopolyploid weed (Capsella bursa-pastoris)
Source: PLoS Genet. 2019 May 13;15(5):e1008131. doi: 10.1371/journal.pgen.1008131 (PMC6532933; doi:10.1371/journal.pgen.1008131)
Supplement: S9 Fig — (PDF) [file pgen.1008131.s009.pdf]

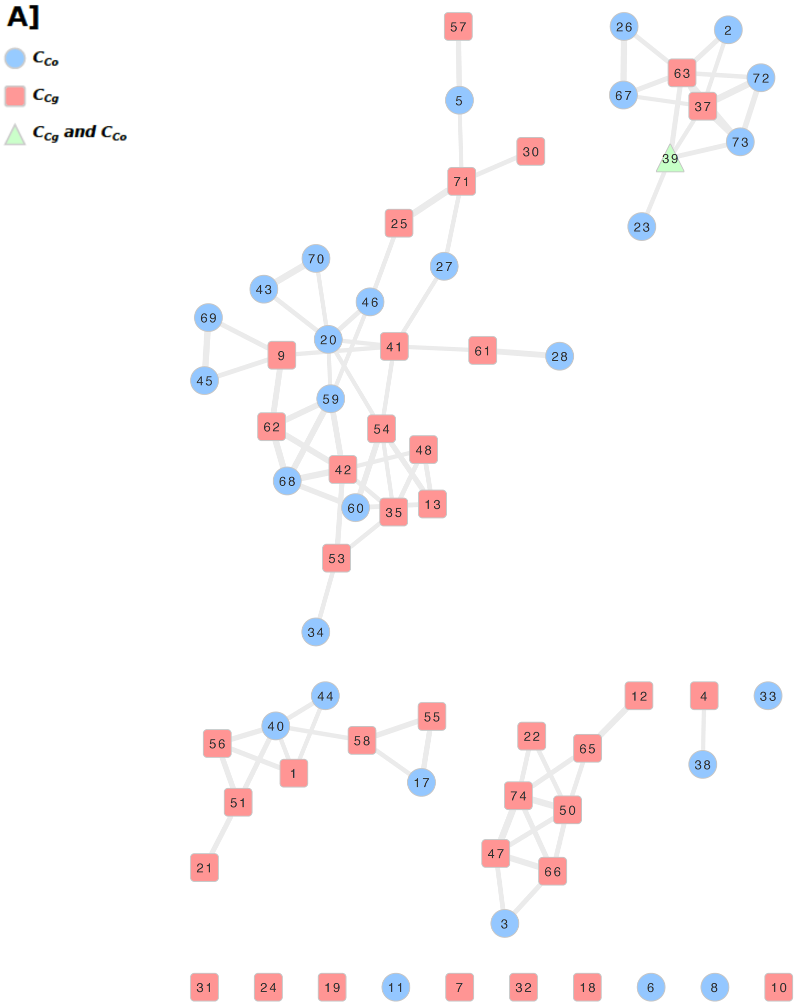

1: response to reactive oxygen species
2: vacuolar transport
3: phloem development
4: cellulose microfibril organization
5: protein peptidyl-prolyl isomerization
6: protein folding
7: regulation of nitrogen utilization
8: carbohydrate metabolism
9: mRNA splicing, via spliceosome
10: primary metabolism
11: nitric oxide biosynthesis
12: cytokinesis by cell plate formation
13: phosphatidylinositol dephosphorylation
14: attachment of GPI anchor to protein
15: ceramide metabolism
16: sphingolipid biosynthesis
17: response to insect
18: L-arabinose metabolism
19: microtubule-based process
20: regulation of translational fidelity
21: regulation of vernalization response
22: vasculature development
23: polyamine transport
24: response to arsenic-containing substance
25: protein deubiquitination
26: retrograde transport, endosome to Golgi
27: protein glycosylation
28: double-strand break repair
29: double-strand break repair via homologous recombination
30: proteolysis
31: response to karrikin
32: response to wounding
33: oxidation-reduction process
34: riboflavin biosynthesis
35: tricarboxylic acid cycle
36: mitochondrial electron transport, ubiquinol to cytochrome c
37: metal ion transport
38: tubulin complex assembly
39: drug transmembrane transport
40: response to auxin
41: regulation of transcription, DNA-templated
42: L-phenylalanine catabolism
43: cell redox homeostasis
44: response to cadmium ion
45: charged-tRNA amino acid modification
46: signal peptide processing
47: procambium histogenesis
48: very long-chain fatty acid metabolism
49: fatty acid biosynthesis
50: vegetative to reproductive phase transition of meristem
51: regulation of auxin mediated signaling pathway
52: auxin-activated signaling pathway
53: glutamate biosynthesis
54: pyrimidine nucleotide biosynthesis
55: response to bacterium
56: brassinosteroid mediated signaling pathway
57: peptidyl-diphthamide biosynthesis
58: response to molecule of bacterial origin
59: allantoin catabolism
60: Mo-molybdopterin cofactor biosynthesis
61: RNA-directed DNA methylation
62: deadenylation-dependent decapping of nuclear-transcribed mRNA
63: transport
64: transmembrane transport
65: male meiosis
66: floral meristem determinacy
67: ER to Golgi vesicle-mediated transport
68: chlorophyll catabolism
69: tRNA threonylcarbamoyladenosine modification
70: regulation of pH
71: protein ubiquitination
72: iron ion transport
73: ATP hydrolysis coupled proton transport
74: embryo development ending in seed dormancy

Continue on the next page

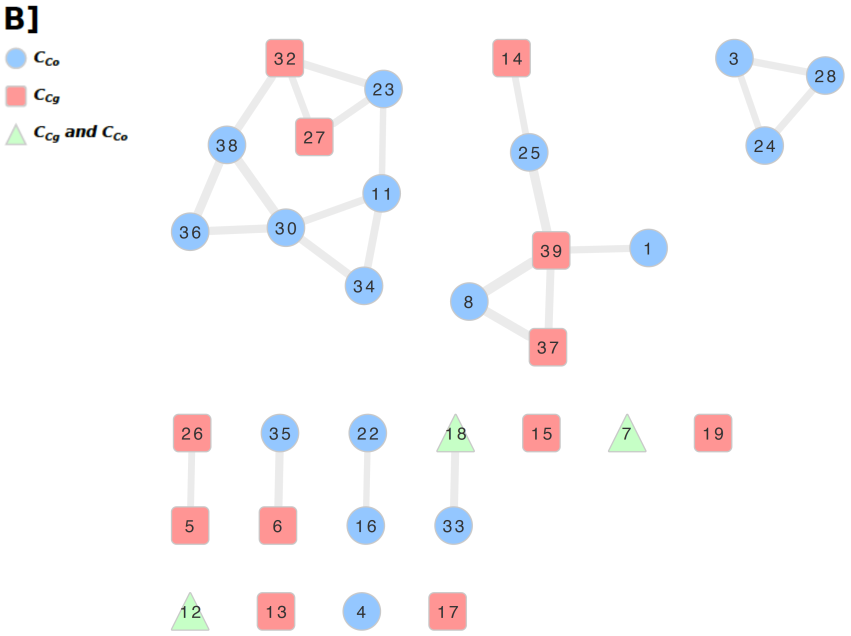

1: nuclear division
2: mitotic sister chromatid segregation
3: drug transmembrane transport
4: metabolism
5: procambium histogenesis
6: regulation of cyclin-dependent protein serine/threonine kinase activity
7: carbohydrate metabolism
8: DNA repair
9: double-strand break repair via homologous recombination
10: base-excision repair
11: cellular amino acid metabolism
12: sucrose metabolism
13: proteolysis
14: lipid storage
15: defense response to oomycetes
16: regulation of auxin mediated signaling pathway
17: response to wounding
18: response to heat
19: response to metal ion
20: response to water deprivation
21: response to cadmium ion
22: response to chitin
23: 'de novo' IMP biosynthesis
24: malate transport
25: cell redox homeostasis
26: vegetative to reproductive phase transition of meristem
27: phosphatidylinositol dephosphorylation
28: potassium ion transmembrane transport
29: potassium ion transport
30: L-phenylalanine catabolism
31: tryptophan biosynthesis
32: Mo-molybdopterin cofactor biosynthesis
33: response to salt stress
34: cysteine biosynthesis from serine
35: cytokinesis by cell plate formation
36: allantoin catabolism
37: RNA-directed DNA methylation
38: chlorophyll catabolism
39: telomere maintenance

Continue on the next page

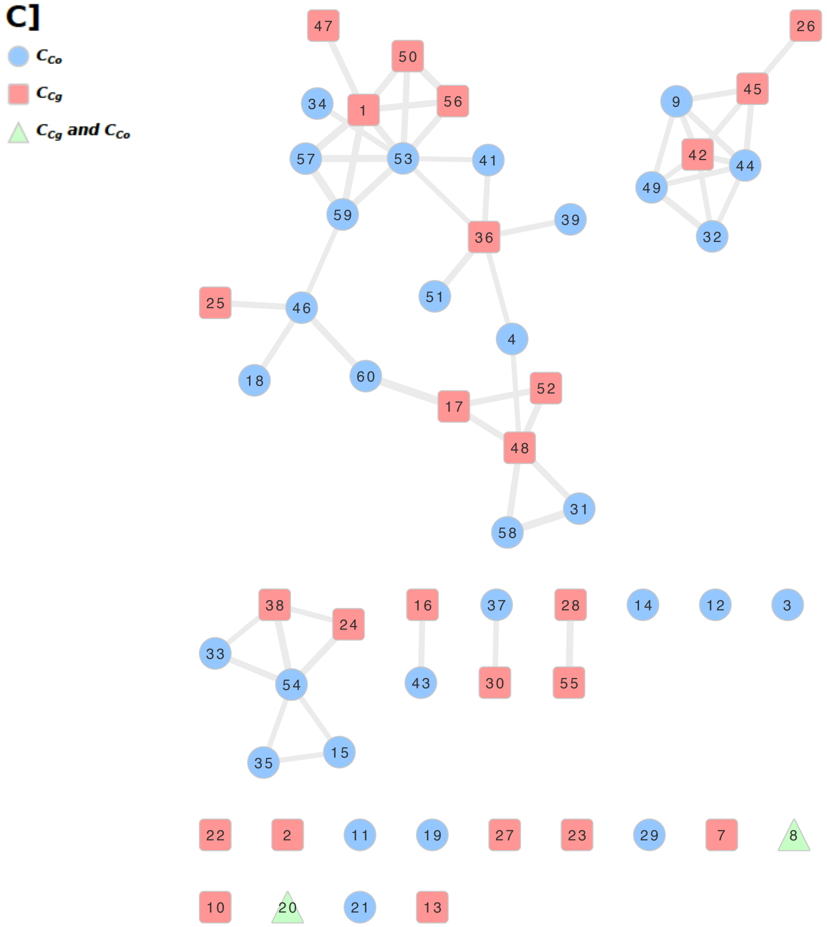

1: GPI anchor biosynthesis
2: metabolism
3: superoxide metabolism
4: cytokinin metabolism
5: tryptophan biosynthesis
6: cytokinin biosynthesis
7: polarity specification of adaxial/abaxial axis
8: carbohydrate metabolism
9: drug transmembrane transport
10: biosynthesis
11: positive regulation of cell proliferation
12: nuclear-transcribed mRNA poly(A) tail shortening
13: tRNA wobble position uridine thiolation
14: microtubule-based process
15: response to salt stress
16: peptidyl-lysine modification to peptidyl-hypusine
17: RNA-directed DNA methylation
18: DNA-templated transcription, initiation
19: phosphorelay signal transduction system
20: proteolysis
21: lipid metabolism
22: phloem or xylem histogenesis
23: regulation of cyclin-dependent protein serine/threonine kinase activity
24: regulation of systemic acquired resistance
25: RNA processing
26: vacuolar transport
27: pollen tube growth
28: xylan biosynthesis
29: aging
30: response to abscisic acid
31: cellular glucose homeostasis
32: basic amino acid transport
33: response to insect
34: deoxyribonucleoside diphosphate metabolism
35: response to oxidative stress
36: cellular amino acid metabolism
37: response to cadmium ion
38: defense response to oomycetes
39: arginine catabolism
40: glutamine metabolism
41: translational initiation
42: potassium ion transmembrane transport
43: protein peptidyl-prolyl isomerization
44: lipid transport
45: transport
46: regulation of transcription, DNA-templated
47: carotenoid biosynthesis
48: telomere maintenance
49: nucleotide-sugar transport
50: thiamine diphosphate biosynthesis
51: salicylic acid metabolism
52: base-excision repair
53: 'de novo' IMP biosynthesis
54: defense response to fungus
55: sucrose metabolism
56: Mo-molybdopterin cofactor biosynthesis
57: protein glycosylation
58: cellular iron ion homeostasis
59: nucleotide-sugar biosynthesis
60: negative regulation of transcription, DNA-templated

Continue on the next page

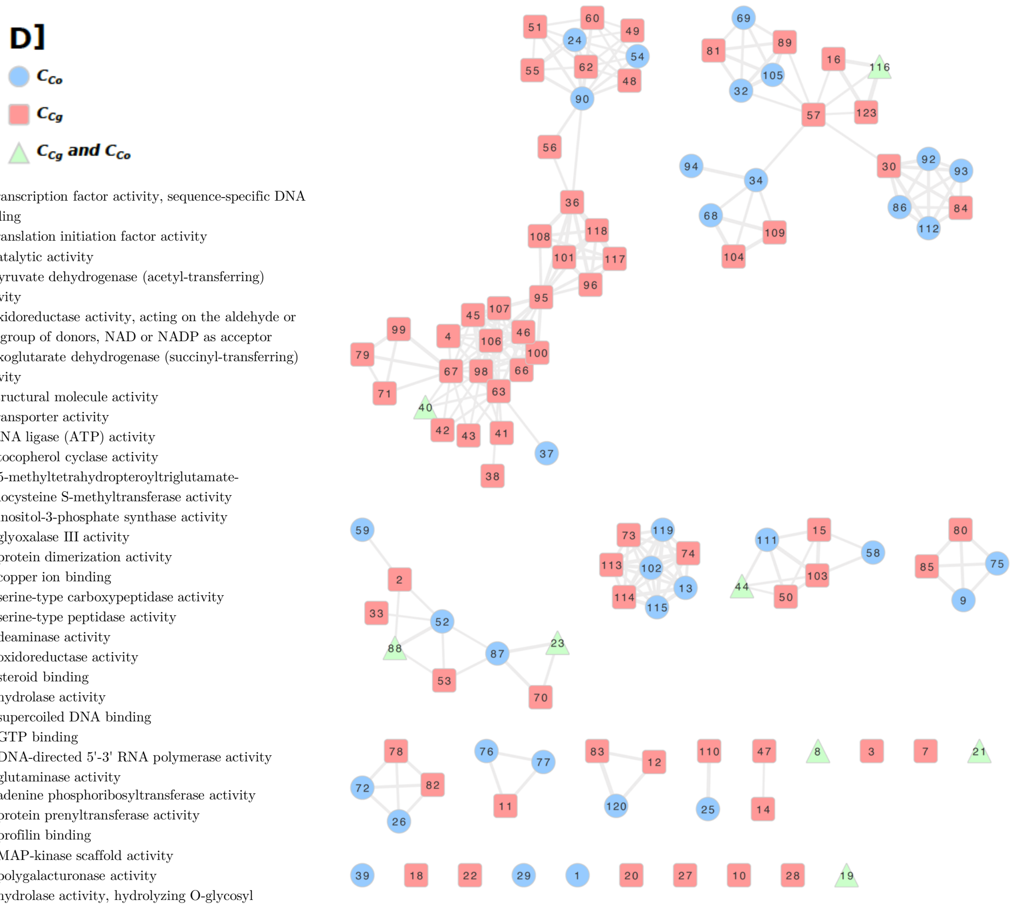

1: transcription factor activity, sequence-specific DNA binding
2: translation initiation factor activity
3: catalytic activity
4: pyruvate dehydrogenase (acetyl-transferring) activity
5: oxidoreductase activity, acting on the aldehyde or oxo group of donors, NAD or NADP as acceptor
6: oxoglutarate dehydrogenase (succinyl-transferring) activity
7: structural molecule activity
8: transporter activity
9: RNA ligase (ATP) activity
10: tocopherol cyclase activity
11: 5-methyltetrahydropteroyltriglutamate-homocysteine S-methyltransferase activity
12: inositol-3-phosphate synthase activity
13: glyoxalase III activity
14: protein dimerization activity
15: copper ion binding
16: serine-type carboxypeptidase activity
17: serine-type peptidase activity
18: deaminase activity
19: oxidoreductase activity
20: steroid binding
21: hydrolase activity
22: supercoiled DNA binding
23: GTP binding
24: DNA-directed 5'-3' RNA polymerase activity
25: glutaminase activity
26: adenine phosphoribosyltransferase activity
27: protein prenyltransferase activity
28: profilin binding
29: MAP-kinase scaffold activity
30: polygalacturonase activity
31: hydrolase activity, hydrolyzing O-glycosyl compounds
32: microtubule motor activity
33: core promoter binding
34: protein serine/threonine phosphatase activity
35: phosphoprotein phosphatase activity
36: transferase activity, transferring acyl groups other than amino-acyl groups
37: homogentisate 1,2-dioxygenase activity
38: L-aspartate oxidase activity
39: ubiquitin binding
40: NADPH-hemoprotein reductase activity
41: ribonucleoside-diphosphate reductase activity, thioredoxin disulfide as acceptor
42: 4-hydroxy-tetrahydrodipicolinate reductase
43: thiol oxidase activity
44: magnesium ion binding
45: UDP-glucose 6-dehydrogenase activity
46: alternative oxidase activity
47: protein domain specific binding
48: glycerol kinase activity
49: thymidylate kinase activity
50: calcium ion binding
51: inositol-1,4,5-trisphosphate 3-kinase activity
52: DNA binding
53: nucleic acid binding
54: diacylglycerol cholinephosphotransferase activity
55: aspartate kinase activity
56: transferase activity, transferring glycosyl groups
57: hydrolase activity, acting on ester bonds
58: molybdenum ion binding
59: mRNA 3'-UTR binding
60: ribokinase activity
61: 6-phosphofructokinase activity
62: 1-phosphatidylinositol 4-kinase activity
63: peroxidase activity
64: catalase activity
65: thioredoxin-disulfide reductase activity
66: squalene monooxygenase activity
67: cytochrome-c oxidase activity
68: pectin acetylesterase activity
69: acylphosphatase activity
70: ADP binding
71: auxin influx transmembrane transporter activity
72: galactoside 2-alpha-L-fucosyltransferase activity
73: N6-(1,2-dicarboxyethyl)AMP AMP-lyase (fumarate-forming) activity
74: uroporphyrinogen decarboxylase activity
75: adenylosuccinate synthase activity
76: 3-methyl-2-oxobutanoate hydroxymethyltransferase activity
77: protein-L-isoaspartate (D-aspartate) O-methyltransferase activity
78: alpha-1,2-mannosyltransferase activity
79: allantoin uptake transmembrane transporter activity
80: NEDD8 activating enzyme activity
81: DNA clamp loader activity
82: galactosylgalactosylxylosylprotein 3-beta-glucuronosyltransferase activity
83: glucose-6-phosphate isomerase activity
84: alpha,alpha-trehalase activity
85: threonine-tRNA ligase activity
86: poly(ADP-ribose) glycohydrolase activity
87: nucleotide binding
88: RNA binding
89: DNA helicase activity
90: protein serine/threonine kinase activity
91: protein kinase activity
92: alpha-L-fucosidase activity
93: beta-N-acetylhexosaminidase activity
94: histidinol-phosphatase activity
95: 3-oxoacyl-[acyl-carrier-protein] reductase (NADPH) activity
96: hydroxymethylglutaryl-CoA synthase activity
97: citrate (Si)-synthase activity
98: D-arabinono-1,4-lactone oxidase activity
99: iron ion transmembrane transporter activity
100: 3-hydroxyacyl-CoA dehydrogenase activity
101: acetyl-CoA C-acetyltransferase activity
102: dihydroneopterin aldolase activity
103: zinc ion binding
104: phospholipase A2 activity
105: GTPase activity
106: glycerol-3-phosphate dehydrogenase [NAD+] activity
107: isocitrate dehydrogenase (NAD+) activity
108: gamma-glutamyltransferase activity
109: aminoacyl-tRNA editing activity
110: asparaginase activity
111: metal ion binding
112: beta-galactosidase activity
113: diphosphomevalonate decarboxylase activity
114: fumarate hydratase activity
115: 4-hydroxy-tetrahydrodipicolinate synthase
116: aspartic-type endopeptidase activity
117: dihydrolipoyllysine-residue acetyltransferase activity
118: peptide alpha-N-acetyltransferase activity
119: tryptophan synthase activity
120: dodecenoyl-CoA delta-isomerase activity
121: protein disulfide isomerase activity
122: isopentenyl-diphosphate delta-isomerase activity
123: metalloendopeptidase activity

Continue on the next page

E]

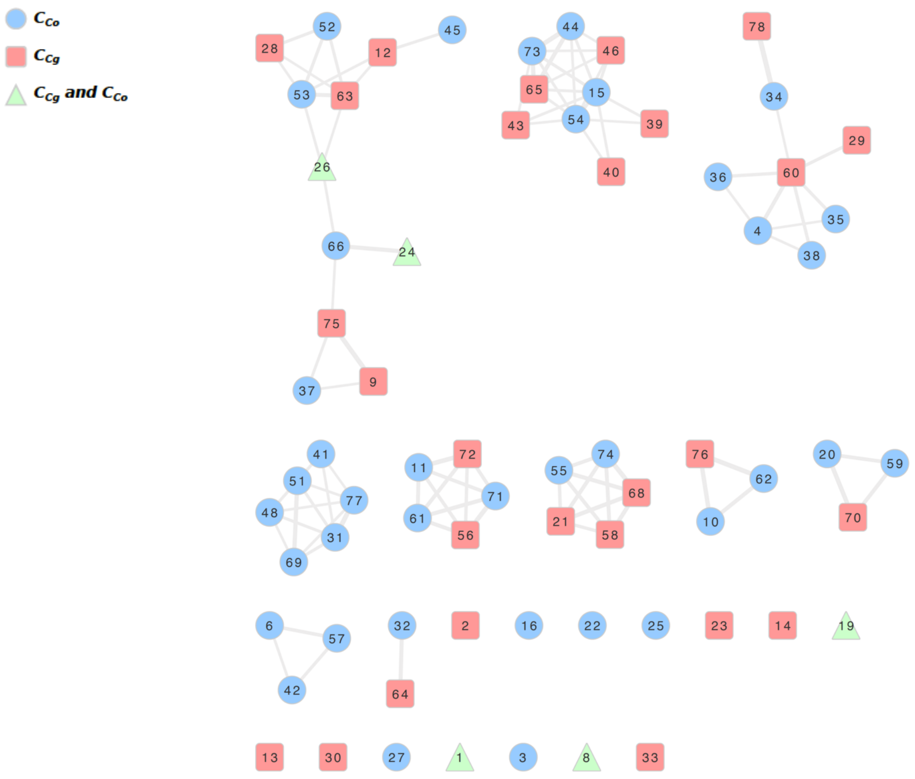

1: transcription factor activity, sequence-specific DNA binding
2: structural constituent of ribosome
3: catalytic activity
4: ribokinase activity
5: galactokinase activity
6: intracellular cAMP activated cation channel activity
7: ionotropic glutamate receptor activity
8: binding
9: iron ion binding
10: chorismate mutase activity
11: glyoxalase III activity
12: translation initiation factor activity
13: threonine-tRNA ligase activity
14: protein dimerization activity
15: peroxidase activity
16: serine-type carboxypeptidase activity
17: serine-type endopeptidase activity
18: omega peptidase activity
19: oxidoreductase activity
20: phosphatidyl-N-methylethanolamine N-methyltransferase activity
21: dolichyl-phosphate-glucose-glycolipid alpha-glucosyltransferase activity
22: transketolase activity
23: thiosulfate sulfurtransferase activity
24: GTP binding
25: profilin binding
26: nucleic acid binding
27: palmitoyl hydrolase activity
28: single-stranded DNA binding
29: ubiquitin-protein transferase activity
30: DNA helicase activity
31: protein serine/threonine phosphatase activity
32: polygalacturonase activity
33: heat shock protein binding
34: glutathione transferase activity
35: phosphomevalonate kinase activity
36: phosphatidate cytidylyltransferase activity
37: magnesium ion binding
38: riboflavin kinase activity
39: dihydroorotate dehydrogenase activity
40: succinate-semialdehyde dehydrogenase [NAD(P)+] activity
41: single-stranded DNA 5'-3' exodeoxyribonuclease activity
42: inorganic phosphate transmembrane transporter activity
43: methylenetetrahydrofolate reductase (NAD(P)H) activity
44: 3-isopropylmalate dehydrogenase activity
45: double-stranded RNA binding
46: peptide-methionine (S)-S-oxide reductase activity
47: peptide-methionine (R)-S-oxide reductase activity
48: tRNA-intron endonuclease activity
49: 3'-5'-exoribonuclease activity
50: ribonuclease III activity
51: methyl jasmonate esterase activity
52: double-stranded DNA binding
53: DNA binding
54: N,N-dimethylaniline monooxygenase activity
55: galactoside 2-alpha-L-fucosyltransferase activity
56: dihydroneopterin aldolase activity
57: magnesium ion transmembrane transporter activity
58: galactosylgalactosylxylosylprotein 3-beta-glucuronosyltransferase activity
59: tRNA methyltransferase activity
60: protein kinase activity
61: carbon-sulfur lyase activity
62: UDP-glucose 4-epimerase activity
63: RNA binding
64: beta-N-acetylhexosaminidase activity
65: long-chain-alcohol oxidase activity
66: ATP binding
67: nucleotide binding
68: cellulose synthase (UDP-forming) activity
69: aminoacyl-tRNA hydrolase activity
70: phosphoethanolamine N-methyltransferase activity
71: glutamate decarboxylase activity
72: 4-hydroxy-tetrahydrodipicolinate synthase
73: phosphogluconate dehydrogenase (decarboxylating) activity
74: glycogen (starch) synthase activity
75: zinc ion binding
76: intramolecular transferase activity
77: nucleotidase activity
78: methionine adenosyltransferase activity

Continue on the next page

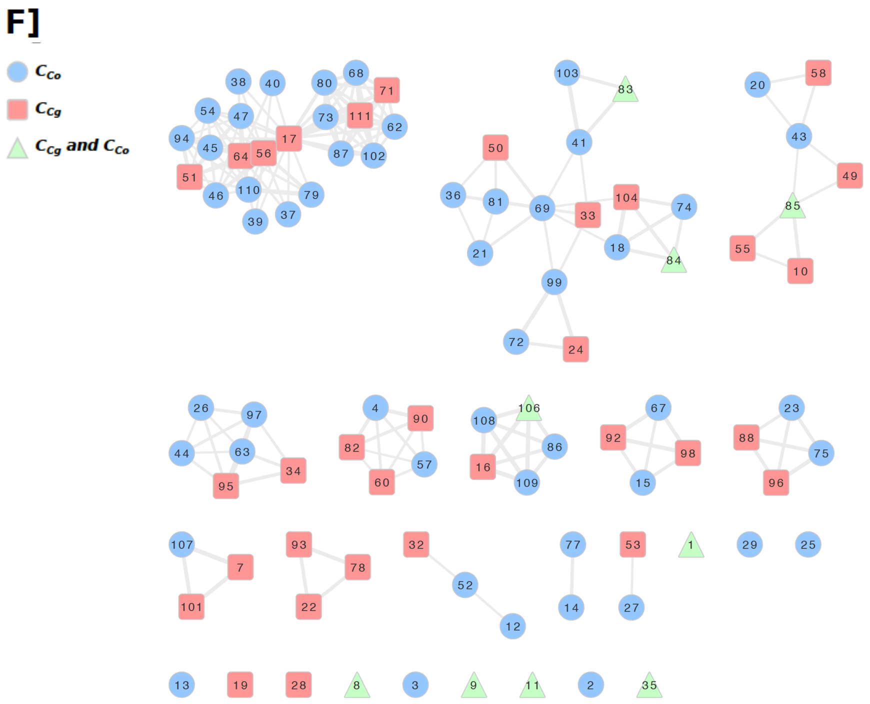

1: transcription factor activity, sequence-specific DNA binding
2: transcription corepressor activity
3: catalytic activity
4: acid phosphatase activity
5: phosphoprotein phosphatase activity
6: protein serine/threonine phosphatase activity
7: GDP-dissociation inhibitor activity
8: transporter activity
9: binding
10: GTP binding
11: electron carrier activity
12: protein heterodimerization activity
13: tocopherol cyclase activity
14: inositol-3-phosphate synthase activity
15: glyoxalase III activity
16: threonine-tRNA ligase activity
17: cytochrome-c oxidase activity
18: O-methyltransferase activity
19: oxidoreductase activity
20: single-stranded DNA binding
21: thymidylate kinase activity
22: AMP deaminase activity
23: 1,2-alpha-L-fucosidase activity
24: 1,4-alpha-glucan branching enzyme activity
25: acetolactate synthase activity
26: calcium ion binding
27: translation release factor activity
28: Ran GTPase binding
29: serine-type endopeptidase activity
30: serine-type carboxypeptidase activity
31: metalloendopeptidase activity
32: actin binding
33: ubiquitin-protein transferase activity
34: manganese ion binding
35: GTPase activity
36: shikimate kinase activity
37: L-lactate dehydrogenase activity
38: sarcosine oxidase activity
39: formate dehydrogenase (NAD+) activity
40: nitrite reductase [NAD(P)H] activity
41: N-acetyltransferase activity
42: acetyl-CoA:L-glutamate N-acetyltransferase activity
43: RNA binding
44: magnesium ion binding
45: NADPH-hemoprotein reductase activity
46: ribonucleoside-diphosphate reductase activity, thioredoxin disulfide as acceptor
47: protochlorophyllide reductase activity
48: 4-hydroxy-tetrahydrodipicolinate reductase
49: nucleic acid binding
50: pantothenate kinase activity
51: peptide-methionine (S)-S-oxide reductase activity
52: protein dimerization activity
53: double-stranded RNA binding
54: alternative oxidase activity
55: NAD binding
56: N,N-dimethylaniline monooxygenase activity
57: pectin acetylesterase activity
58: transcription regulatory region sequence-specific DNA binding
59: core promoter proximal region sequence-specific DNA binding
60: 3'-5'-exoribonuclease activity
61: ribonuclease III activity
62: UDP-galactose transmembrane transporter activity
63: copper ion binding
64: peroxidase activity
65: catalase activity
66: superoxide dismutase activity
67: 1,4-dihydroxy-2-naphthoyl-CoA synthase activity
68: voltage-gated chloride channel activity
69: protein serine/threonine kinase activity
70: protein kinase activity
71: protein transporter activity
72: galactoside 2-alpha-L-fucosyltransferase activity
73: allantoin uptake transmembrane transporter activity
74: aspartate carbamoyltransferase activity
75: polygalacturonase activity
76: hydrolase activity, hydrolyzing O-glycosyl compounds
77: ribose-5-phosphate isomerase activity
78: beta-ureidopropionase activity
79: nitronate monooxygenase activity
80: ammonium transmembrane transporter activity
81: NAD+ kinase activity
82: histidinol-phosphatase activity
83: arginyltransferase activity
84: 3-methyl-2-oxobutanoate hydroxymethyltransferase activity
85: nucleotide binding
86: glutamate-ammonia ligase activity
87: inorganic phosphate transmembrane transporter activity
88: alpha-amylase activity
89: beta-amylase activity
90: phosphatidylinositol phospholipase C activity
91: phospholipase D activity
92: adenosylmethionine decarboxylase activity
93: asparaginase activity
94: thiol oxidase activity
95: zinc ion binding
96: chitinase activity
97: metal ion binding
98: dihydroneopterin aldolase activity
99: UDP-glucosyltransferase activity
100: cellulose synthase (UDP-forming) activity
101: enzyme inhibitor activity
102: intracellular cAMP activated cation channel activity
103: diacylglycerol O-acyltransferase activity
104: histone-lysine N-methyltransferase activity
105: protein-L-isoaspartate (D-aspartate) O-methyltransferase activity
106: cysteine-tRNA ligase activity
107: Rab GDP-dissociation inhibitor activity
108: glycine-tRNA ligase activity
109: proline-tRNA ligase activity
110: acireductone dioxygenase [iron(II)-requiring] activity
111: potassium ion transmembrane transporter activity
112: iron ion transmembrane transporter activity

Fig. S9. Network shared names of enriched biological processes (A, B and C) or molecular functions (D, E and F) GO term for genes showing convregence in expression between subgenomes in flowers (A and D), leaves (B and E) or roots tissues (C and F). Co indicates a convergence of Cbp_{Cg} toward Cbp_{Co}, and Cg corresponds to a convergence of Cbp_{Co} toward Cbp_{Cg}.
