# Supplementary material for: Towards the new normal: Transcriptomic convergence and genomic legacy of the two subgenomes of an allopolyploid weed (Capsella bursa-pastoris)
Source: PLoS Genet. 2019 May 13;15(5):e1008131. doi: 10.1371/journal.pgen.1008131 (PMC6532933; doi:10.1371/journal.pgen.1008131)
Supplement: S10 Fig — (PDF) [file pgen.1008131.s010.pdf]

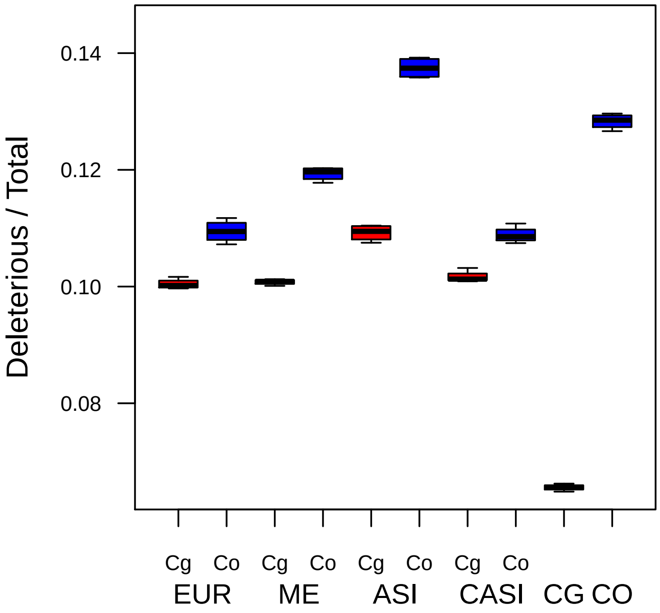

Fig. S10. Proportion of deleterious mutations in the two subgenomes of *C. bursa-pastoris* and the genomes of its parental species. CO, CG, ASI, EUR, ME, CASI correspond to *C. orientalis*, *C. grandiflora*, and four populations of *C. bursa-pastoris*, respectively. The two subgenomes are indicated with Co and Cg. Functional effects were annotated with the *A. thaliana* SIFT database.
