# Supplementary material for: Towards the new normal: Transcriptomic convergence and genomic legacy of the two subgenomes of an allopolyploid weed (Capsella bursa-pastoris)
Source: PLoS Genet. 2019 May 13;15(5):e1008131. doi: 10.1371/journal.pgen.1008131 (PMC6532933; doi:10.1371/journal.pgen.1008131)
Supplement: S11 Fig — (PDF) [file pgen.1008131.s011.pdf]

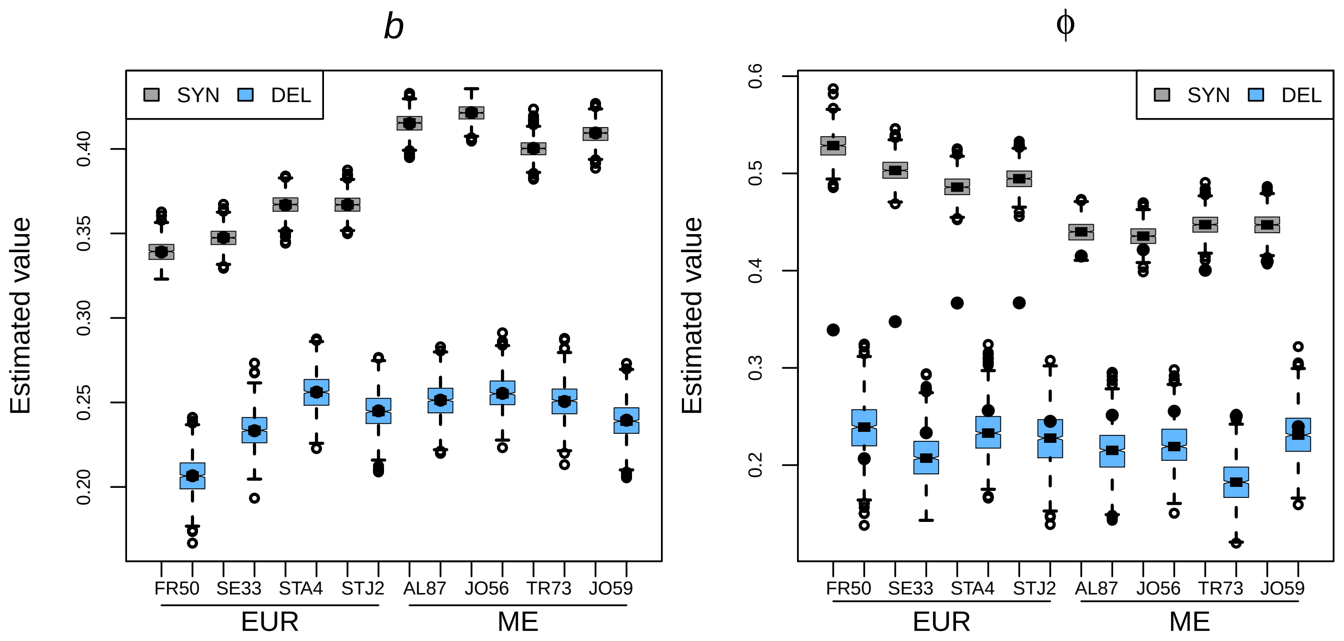

Fig. S11. Maximum likelihood estimated parameters of the distribution of deleterious mutations on Cbp_{Cg} genes. Each box represents the estimates of one accession, with 1000 bootstrap replicates. The estimated parameters are for synonymous mutations (SYN), and deleterious mutations (DEL). The notch of the plot represents the median and the 95% confidence interval. The black points are the point estimations with the original samples, instead of the bootstrap re-samples. The left figure shows estimates of the bias parameter, b, and the right figure shows estimates of the variation parameter φ.
