# Supplementary figures and images for: Towards the new normal: Transcriptomic convergence and genomic legacy of the two subgenomes of an allopolyploid weed (Capsella bursa-pastoris)
Source: PLoS Genet. 2019 May 13;15(5):e1008131. doi: 10.1371/journal.pgen.1008131 (PMC6532933; doi:10.1371/journal.pgen.1008131)

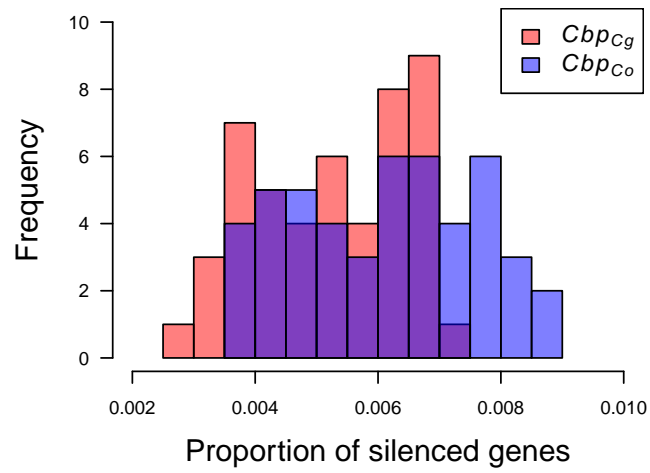

Fig. S12. The difference in the number of silenced genes between subgenomes of *C. bursa-pastoris*.

Supplement: S12 Fig — (PDF) [file pgen.1008131.s012.pdf]
