# Supplementary material for: Towards the new normal: Transcriptomic convergence and genomic legacy of the two subgenomes of an allopolyploid weed (Capsella bursa-pastoris)
Source: PLoS Genet. 2019 May 13;15(5):e1008131. doi: 10.1371/journal.pgen.1008131 (PMC6532933; doi:10.1371/journal.pgen.1008131)
Supplement: S1 Table — (PDF) [file pgen.1008131.s013.pdf]

Accession	Population	Latitude	Longitude	DNA lib.size	Flower RNA lib.size	Leaf RNA lib.size	Root RNA lib.size	DNA-Seq (SRA)	Flower RNA-Seq (SRA)	Leaf RNA-Seq (SRA)	Root RNA-Seq (SRA)
DL174	ASI	38.56	121.35	76005581	74101388	65425962	51624022	SRS2762453	SRR8944199	SRR8944136	SRR8944193
JZH152	ASI	30.20	112.06	80652085	61417236	61334938	37507282	SRS2762444	SRR8944198	SRR8944137	SRR8944192
NJ219	ASI	32.03	118.46	67776611	62976278	87650066	46483576	SRS2762448	SRR8944172	SRR8944134	SRR8944191
TY118	ASI	37.55	112.32	68816910	53541728	60561340	58656088	SRS2762440	SRR8944166	SRR8944135	SRR8944190
DUB-RUS9	CASI	51.53	58.85	84128727	78319556	68099236	54375028	SRR8904462	SRR8944201	SRR8944151	SRR8944130
KYRG-3-14	CASI	39.79	72.18	45154567	66059762	60694436	57074026	SRR8904463	SRR8944200	SRR8944152	SRR8944133
LAB-RUS-4	CASI	66.65	66.40	33843140	69017208	61974690	39439188	SRR8904464	SRR8944181	SRR8944157	SRR8944148
TACH-CHIN14	CASI	47.07	83.01	83324082	61379494	77626154	38668184	SRR8904465	SRR8944184	SRR8944158	SRR8944149
85.3	CG	39.56	20.92	57969763	55106794	68729596	59193184	SRR8904467	SRR8944183	SRR8944155	SRR8944173
86.12	CG	39.52	20.97	71852215	70392238	54187192	62579512	SRR8904468	SRR8944182	SRR8944156	SRR8944169
87.26	CG	39.48	20.98	60716530	62728886	59460660	64762960	SRR8904469	SRR8944195	SRR8944146	SRR8944177
88.5	CG	39.88	20.75	45369106	67964672	44978638	47326734	SRR8904470	SRR8944178	SRR8944147	SRR8944175
GUB-RUS5	CO	51.29	58.18	55352931	58041160	55549182	61419992	SRR8904471	SRR8944179	SRR8944189	SRR8944174
PAR-RUS	CO	53.30	60.10	49028787	68030766	55527408	57415936	SRR8904459	SRR8944144	SRR8944188	SRR8944150
QH-CHIN4	CO	46.70	90.83	46773214	66241216	55334438	57763206	SRR8904460	SRR8944167	SRR8944187	SRR8944165
URAL-RUS4	CO	55.11	61.39	45387760	76656450	57886342	69234050	SRR8904461	SRR8944180	SRR8944186	SRR8944159
FR50	EUR	48.08	7.37	70612809	57720098	46159028	33748500	SRS2762459	SRR8944163	SRR8944140	SRR8944197
SE33	EUR	56.15	13.77	78120829	61139124	66478952	49599858	SRS2762447	SRR8944164	SRR8944141	SRR8944196
STA4	EUR	56.20	-2.47	76063405	64453046	52318632	42635412	SRS2762460	SRR8944161	SRR8944176	SRR8944138
STJ2	EUR	44.51	-1.21	77687488	85809658	48134448	35801122	SRS2762442	SRR8944162	SRR8944139	SRR8944142
AL87	ME	35.45	7.96	59970536	76995752	57011620	54660030	SRS2762455	SRR8944168	SRR8944131	SRR8944143
JO56	ME	31.97	35.98	79470603	56584612	61885264	60461952	SRS2762457	SRR8944160	SRR8944132	SRR8944145
TR73	ME	41.02	28.97	77624846	59569756	69614876	44957844	SRS2762445	SRR8944170	SRR8944153	SRR8944185
JO59	ME	31.97	35.98	67354447	67068132	68539844	53689276	SRR8904466	SRR8944171	SRR8944154	SRR8944194
