# Supplementary material for: Towards the new normal: Transcriptomic convergence and genomic legacy of the two subgenomes of an allopolyploid weed (Capsella bursa-pastoris)
Source: PLoS Genet. 2019 May 13;15(5):e1008131. doi: 10.1371/journal.pgen.1008131 (PMC6532933; doi:10.1371/journal.pgen.1008131)
Supplement: S2 Table — (PDF) [file pgen.1008131.s014.pdf]

Table S2. Differential gene expression between three *Capsella* species in three tissues.

Tissue	Comparison		
	CO vs CG	Cbp vs CG	Cbp vs CO
Flowers	4793	4214	**2796**
Leaves	1729	**1172**	1535
Roots	2742	**2047**	3537

CO, CG, and Cbp correspond to *C. orientalis*, *C. grandiflora*, and *C. bursa-pastoris*, respectively. The analysis was performed on the unphased expression data of 16,032 genes with the significance level set to 0.05. The smallest differences between species per tissue are in bold.
