# Supplementary material for: Towards the new normal: Transcriptomic convergence and genomic legacy of the two subgenomes of an allopolyploid weed (Capsella bursa-pastoris)
Source: PLoS Genet. 2019 May 13;15(5):e1008131. doi: 10.1371/journal.pgen.1008131 (PMC6532933; doi:10.1371/journal.pgen.1008131)
Supplement: S3 Table — (PDF) [file pgen.1008131.s015.pdf]

Table S3. Differential gene expression between *Capsella* species/population in three tissues.

Tissue	Comparison															
	CG vs ASI	CG vs EUR	CG vs ME	CG vs CASI	CO vs ASI	CO vs EUR	CO vs ME	CO vs CASI	ASI vs EUR	ASI vs ME	EUR vs ME	ASI vs CASI	EUR vs CASI	ME vs CASI
Flowers	3946	3677	3723	**3395**	2467	3069	2358	**2084**	907	908	680	645	**62**	458
Leaves	**851**	1192	1031	1055	1154	1392	**978**	1612	394	551	245	360	**7**	212
Roots	1868	2123	**1533**	1592	2167	4000	**2138**	3305	847	1052	514	570	**7**	358

CO, CG, ASI, CASI, EUR, and ME correspond to *C. orientalis*, *C. grandiflora*, and four populations of *C. bursa-pastoris*, respectively. The analysis was performed on the unphased expression data of 16,032 genes with the significance level set to 0.05. The smallest differences between species/population per tissue are in bold.
