# Supplementary material for: Towards the new normal: Transcriptomic convergence and genomic legacy of the two subgenomes of an allopolyploid weed (Capsella bursa-pastoris)
Source: PLoS Genet. 2019 May 13;15(5):e1008131. doi: 10.1371/journal.pgen.1008131 (PMC6532933; doi:10.1371/journal.pgen.1008131)
Supplement: S4 Table — (PDF) [file pgen.1008131.s016.pdf]

Table S4. Expression ratio between the two subgenomes of *C. bursa-pastoris* across populations in three tissues.

Tissue	Population	All genes	Significant HSE	Deviance
flowers	ASI	0.498	0.497	0.001
flowers	EUR	0.497	0.494	0.002
flowers	ME	0.495	0.491	0.004
leaves	ASI	0.498	0.496	0.002
leaves	EUR	0.496	0.491	0.005
leaves	ME	0.495	0.489	0.006
roots	ASI	0.498	0.497	0.001
roots	EUR	0.497	0.493	0.003
roots	ME	0.495	0.490	0.005

The expression ratio is estimated by the proportion of the Cbp_{Co} subgenome counts in the total expression counts. The table shows ratios for all assayed genes (All genes), genes with significant homeologue-specific expression (Significant HSE), over Asian (ASI), European (EUR) and Middle Eastern (ME) populations of *C. bursa-pastoris* in three different tissues. The deviance shows the difference in mean expression ration between all genes and genes showing significant HSE.
