# Supplementary material for: Towards the new normal: Transcriptomic convergence and genomic legacy of the two subgenomes of an allopolyploid weed (Capsella bursa-pastoris)
Source: PLoS Genet. 2019 May 13;15(5):e1008131. doi: 10.1371/journal.pgen.1008131 (PMC6532933; doi:10.1371/journal.pgen.1008131)
Supplement: S5 Table — (PDF) [file pgen.1008131.s017.pdf]

Table S5. Differentially expressed genes between tissues (A) and populations within tissues (B) for each *C. bursa-pastoris* subgenomes.

A

Contrast	*Cbp*$_{Co}$	%	*Cbp*$_{Cg}$	%	Δ%
F vs L	**6474**	**54**	**6475**	**54**	0
F *vs* R	5922	50	6003	50	-1
L *vs* R	*5545*	*46*	*5688*	*48*	-1

F: Flowers, L: leaves and R: Roots. *FDR* < 0.05.

B

Flowers: 4029					
**Contrast**	***Cbp***$_{Co}$	**%**	***Cbp***$_{Cg}$	**%**	Δ%
ME *vs* ASI	**279**	**7**	223	6	1
ME *vs* CASI	214	5	182	5	1
ME *vs* EUR	216	5	182	5	1
ASI *vs* CASI	192	5	198	5	0
ASI *vs* EUR	268	7	**259**	**6**	0
CASI *vs* EUR	*88*	*2*	*112*	*3*	-1
**Leaves: 3051**					
**Contrast**	***Cbp***$_{Co}$	**%**	***Cbp***$_{Cg}$	**%**	Δ%
ME *vs* ASI	**248**	**8**	**236**	**8**	0
ME *vs* CASI	162	5	134	4	1
ME *vs* EUR	132	4	133	4	0
ASI *vs* CASI	170	6	177	6	0
ASI *vs* EUR	156	5	168	6	0
CASI *vs* EUR	*14*	*0*	*20*	*1*	0
**Roots: 3759**					
**Contrast**	***Cbp***$_{Co}$	**%**	***Cbp***$_{Cg}$	**%**	Δ%
ME *vs* ASI	**256**	**7**	**243**	**6**	0
ME *vs* CASI	137	4	125	3	0
ME *vs* EUR	212	6	180	5	1
ASI *vs* CASI	165	4	174	5	0
ASI *vs* EUR	251	7	241	6	0
CASI *vs* EUR	*23*	*1*	*34*	*1*	0

ASI: Asia, CASI: central Asia, EUR: Europe and ME: Middle-East. *FDR* < 0.05.
