# Supplementary material for: Towards the new normal: Transcriptomic convergence and genomic legacy of the two subgenomes of an allopolyploid weed (Capsella bursa-pastoris)
Source: PLoS Genet. 2019 May 13;15(5):e1008131. doi: 10.1371/journal.pgen.1008131 (PMC6532933; doi:10.1371/journal.pgen.1008131)
Supplement: S6 Table — (PDF) [file pgen.1008131.s018.pdf]

Table S6. Overlap between expression profiles in gene ontology term enrichment for Biological processes (A) and Molecular functions (B).

A

Biological processes Flowers							
Categories	Comp. Drift	Dom. CG	Dom. CO	Int.	Leg.	Rev.	Trans.
Dominance CG	0	-	-	-	-	-	-
Dominance CO	12	13	-	-	-	-	-
Intermediate	12	0	3	-	-	-	-
Legacy	9	3	9	17	-	-	-
Reverse	0	0	0	6	3	-	-
Transgressive	9	2	5	7	12	2	-
No difference	3	3	4	6	3	1	8

Biological processes Leaves							
Categories	Comp. Drift	Dom. CG	Dom. CO	Int.	Leg.	Rev.	Trans.
Dominance CG	19	-	-	-	-	-	-
Dominance CO	0	0	-	-	-	-	-
Intermediate	14	14	0	-	-	-	-
Legacy	14	5	5	18	-	-	-
Reverse	0	0	0	0	0	-	-
Transgressive	3	0	9	0	3	0	-
No difference	4	5	2	5	3	0	5

Biological processes Roots							
Categories	Comp. Drift	Dom. CG	Dom. CO	Int.	Leg.	Rev.	Trans.
Dominance CG	11	-	-	-	-	-	-
Dominance CO	0	7	-	-	-	-	-
Intermediate	9	7	9	-	-	-	-
Legacy	14	11	5	9	-	-	-
Reverse	0	0	0	5	5	-	-
Transgressive	6	10	8	10	6	2	-
No difference	3	6	2	5	1	1	11

B

Molecular functions Flowers							
Categories	Comp. Drift	Dom. CG	Dom. CO	Int.	Leg.	Rev.	Trans.
Dominance CG	4	-	-	-	-	-	-
Dominance CO	11	5	-	-	-	-	-
Intermediate	14	7	10	-	-	-	-
Legacy	16	7	19	21	-	-	-
Reverse	5	12	6	5	4	-	-
Transgressive	17	4	14	11	14	1	-
No difference	9	1	7	1	10	1	7

Molecular functions Leaves							
Categories	Comp. Drift	Dom. CG	Dom. CO	Int.	Leg.	Rev.	Trans.
Dominance CG	6	-	-	-	-	-	-
Dominance CO	0	21	-	-	-	-	-
Intermediate	10	10	8	-	-	-	-
Legacy	12	12	12	18	-	-	-
Reverse	3	14	15	4	7	-	-
Transgressive	8	8	2	20	8	3	-
No difference	3	5	3	3	7	2	2

Molecular functions Roots							
Categories	Comp. Drift	Dom. CG	Dom. CO	Int.	Leg.	Rev.	Trans.
Dominance CG	5	-	-	-	-	-	-
Dominance CO	5	11	-	-	-	-	-
Intermediate	13	12	10	-	-	-	-
Legacy	14	20	7	17	-	-	-
Reverse	7	3	4	5	2	-	-
Transgressive	13	8	8	14	9	8	-
No difference	6	7	4	3	4	1	12
