# Supplementary material for: Towards the new normal: Transcriptomic convergence and genomic legacy of the two subgenomes of an allopolyploid weed (Capsella bursa-pastoris)
Source: PLoS Genet. 2019 May 13;15(5):e1008131. doi: 10.1371/journal.pgen.1008131 (PMC6532933; doi:10.1371/journal.pgen.1008131)
Supplement: S7 Table — (PDF) [file pgen.1008131.s019.pdf]

Table S7. Overlap between tissue in expression profiles gene ontology term enrichment for Biological processes (A) and Molecular functions (B).

A

Categories	Biological processes				
	F *vs* L	F *vs* R	L *vs* R	F *vs* L *vs* R	Single
Reverse	0	22	11	0	67
Transgressive	3	14	10	4	69
Legacy	2	9	7	2	80
Dominance CG	8	8	3	0	81
Comp. drift	5	7	2	0	86
Intermediate	3	2	5	2	88
Dominance CO	3	6	0	0	91
Average	3	10	5	1	80

B

Categories	Molecular functions				
	F *vs* L	F *vs* R	L *vs* R	F *vs* L *vs* R	Single
Transgressive	2	15	4	7	72
Reverse	0	10	10	5	75
Intermediate	2	9	8	3	78
Legacy	5	11	3	2	79
Dominance CG	5	7	8	0	80
Comp. Drift	4	7	6	2	81
Dominance CO	4	5	1	4	86
Average	3	9	6	3	79
