# Supplementary material for: Towards the new normal: Transcriptomic convergence and genomic legacy of the two subgenomes of an allopolyploid weed (Capsella bursa-pastoris)
Source: PLoS Genet. 2019 May 13;15(5):e1008131. doi: 10.1371/journal.pgen.1008131 (PMC6532933; doi:10.1371/journal.pgen.1008131)
Supplement: S8 Table — (PDF) [file pgen.1008131.s020.pdf]

Table S8. Contingency table of number of genes per category based on deleterious mutation and homelogue expression bias.

Mutation types	***Tissues***		***Obs. (Exp.)***		**Total**	***p***
			$e > 0.5$	$e < 0.5$		
DEL	Flowers	$d > 0$	5422 (5118)	5126 (5430)	10548	< 2.2E-16
		$d < 0$	2656 (2960)	3443 (3139)	6099	
		Total	8078	8569	16647	
	Leaves	$d > 0$	5666 (5427)	5749 (5988)	11415	1.1E-13
		$d < 0$	2862 (3101)	3660 (3421)	6522	
		Total	8528	9409	17937	
	Roots	$d > 0$	5299 (5128)	5280 (5451)	10579	2.1E-08
		$d < 0$	2638 (2809)	3158 (2987)	5796	
		Total	7937	8438	16375	
SYN	Flowers	$d > 0$	9857 (9837)	10864 (10884)	20721	0.58
		$d < 0$	2907 (2927)	3258 (3238)	6165	
		Total	12764	14122	26886	
	Leaves	$d > 0$	10189 (10201)	11432 (11420)	21621	0.74
		$d < 0$	3004 (2992)	3338 (3350)	6342	
		Total	13193	14770	27963	
	Roots	$d > 0$	9394 (9431)	10371 (10334)	19765	0.27
		$d < 0$	2848 (2811)	3042 (3079)	5890	
		Total	12242	13413	25655	

d is the difference of number of mutations (DEL, deleterious and SYN, synonymous) between homeologous copies (DEL_{Cg} - DEL_{Co} or SYN_{Cg} - SYN_{Co}) and *e* is the expression ratio between the two homeologues copies with significant HSE ($e = \frac{Cbp_{Co}}{Cbp_{Cg} + Cbp_{Co}}$). For each category, expected number of gene under category independency hypothesis are given into parenthesis, *p* is the *p*-value of Fisher's exact test of independency.
