# Supplementary material for: Towards the new normal: Transcriptomic convergence and genomic legacy of the two subgenomes of an allopolyploid weed (Capsella bursa-pastoris)
Source: PLoS Genet. 2019 May 13;15(5):e1008131. doi: 10.1371/journal.pgen.1008131 (PMC6532933; doi:10.1371/journal.pgen.1008131)
Supplement: S9 Table — (PDF) [file pgen.1008131.s021.pdf]

Table S9. Gene expression levels in *C. bursa-pastoris* and its parental species with different FDR thresholds

Expression pattern	Flower		Leaf		Root	
FDR	0.01	0.1	0.01	0.1	0.01	0.1
No difference 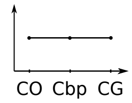	11 079 (70.0%)	7 354 (46.5%)	14 164 (89.5%)	11 391 (72.0%)	12 536 (79.2%)	8 750 (55.3%)
Additivity 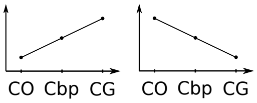	1 157 (7.3 %)	1 759 (11.1%)	450 (2.8%)	877 (5.5%)	638 (4.0%)	1 077 (6.8%)
Dominance 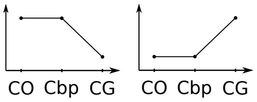	1 435 (9.1%)	2 600 (16.4%)	249 (1.6%)	747 (4.7%)	383 (2.4%)	955 (6.0%)
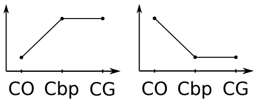	712 (4.5%)	1 551 (9.8%)	365 (2.3%)	997 (6.3%)	714 (4.5%)	1 731 (10.9%)
Transgressive 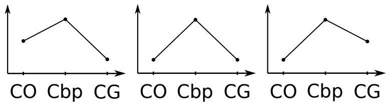	738 (4.7%)	1370 (8.7%)	282 (1.8%)	850 (5.4%)	718 (4.5%)	1 560 (9.9%)
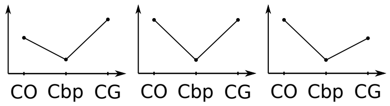	703 (4.4%)	1 190 (7.5%)	314 (2.0%)	962 (6.1%)	835 (5.3%)	1 751 (11.1%)

CO, CG and Cbp correspond to *C. orientalis*, *C. grandiflora*, and *C. bursa-pastoris*, respectively. The levels of expression were considered different if they showed significant differential expression at 0.01 and 0.1 FDR level.
